# Potential Therapeutic Targets and Vaccine Development for SARS-CoV-2/COVID-19 Pandemic Management: A Review on the Recent Update

**DOI:** 10.3389/fimmu.2021.658519

**Published:** 2021-06-30

**Authors:** Uttpal Anand, Shweta Jakhmola, Omkar Indari, Hem Chandra Jha, Zhe-Sheng Chen, Vijay Tripathi, José M. Pérez de la Lastra

**Affiliations:** ^1^ Department of Life Sciences, National Institute for Biotechnology in the Negev, Ben-Gurion University of the Negev, Beer-Sheva, Israel; ^2^ Discipline of Biosciences and Biomedical Engineering, Indian Institute of Technology Indore, Indore, India; ^3^ Department of Pharmaceutical Sciences, College of Pharmacy and Health Sciences, St. John’s University, Queens, NY, United States; ^4^ Department of Molecular and Cellular Engineering, Jacob Institute of Biotechnology and Bioengineering, Sam Higginbottom University of Agriculture, Technology and Sciences, Prayagraj, India; ^5^ Instituto de Productos Naturales y Agrobiología (IPNA), Consejo Superior de Investigaciones científicas (CSIS), Santa Cruz de Tenerife, Spain

**Keywords:** COVID-19, vaccine, therapeutic targets, peptide, antibodies, treatment

## Abstract

Severe acute respiratory syndrome coronavirus-2 (SARS-CoV-2) is a highly pathogenic novel virus that has caused a massive pandemic called coronavirus disease 2019 (COVID-19) worldwide. Wuhan, a city in China became the epicenter of the outbreak of COVID-19 in December 2019. The disease was declared a pandemic globally by the World Health Organization (WHO) on 11 March 2020. SARS-CoV-2 is a beta CoV of the *Coronaviridae* family which usually causes respiratory symptoms that resemble common cold. Multiple countries have experienced multiple waves of the disease and scientific experts are consistently working to find answers to several unresolved questions, with the aim to find the most suitable ways to contain the virus. Furthermore, potential therapeutic strategies and vaccine development for COVID-19 management are also considered. Currently, substantial efforts have been made to develop successful and safe treatments and SARS-CoV-2 vaccines. Some vaccines, such as inactivated vaccines, nucleic acid-based, and vector-based vaccines, have entered phase 3 clinical trials. Additionally, diverse small molecule drugs, peptides and antibodies are being developed to treat COVID-19. We present here an overview of the virus interaction with the host and environment and anti-CoV therapeutic strategies; including vaccines and other methodologies, designed for prophylaxis and treatment of SARS-CoV-2 infection with the hope that this integrative analysis could help develop novel therapeutic approaches against COVID-19.

## Introduction

Several viral diseases have challenged humanity since the beginning of the 21^st^ century, presenting new obstacles to the global public health services. In December 2019, a beta coronavirus (CoV) was identified, now known as the severe acute respiratory syndrome coronavirus-2 (SARS-CoV-2), which is currently greatly threatening the lives of people all over the globe. The current pandemic demands an urgent call for the discovery of new potential molecules or vaccines to fight SARS-CoV-2. In this review, we present a detailed perspective of SARS-CoV-2, from its origin, related viruses, with special emphasis on transmission from environment to human, host-virus interaction, and mechanism of action. We also summarize the status of currently available pharmaceuticals for the treatment of COVID-19 and vaccines, which are being under various stages of clinical trials.

## The Integral Members of Coronavirus Family, Their History, and Associated Diseases

**Figure 1:**
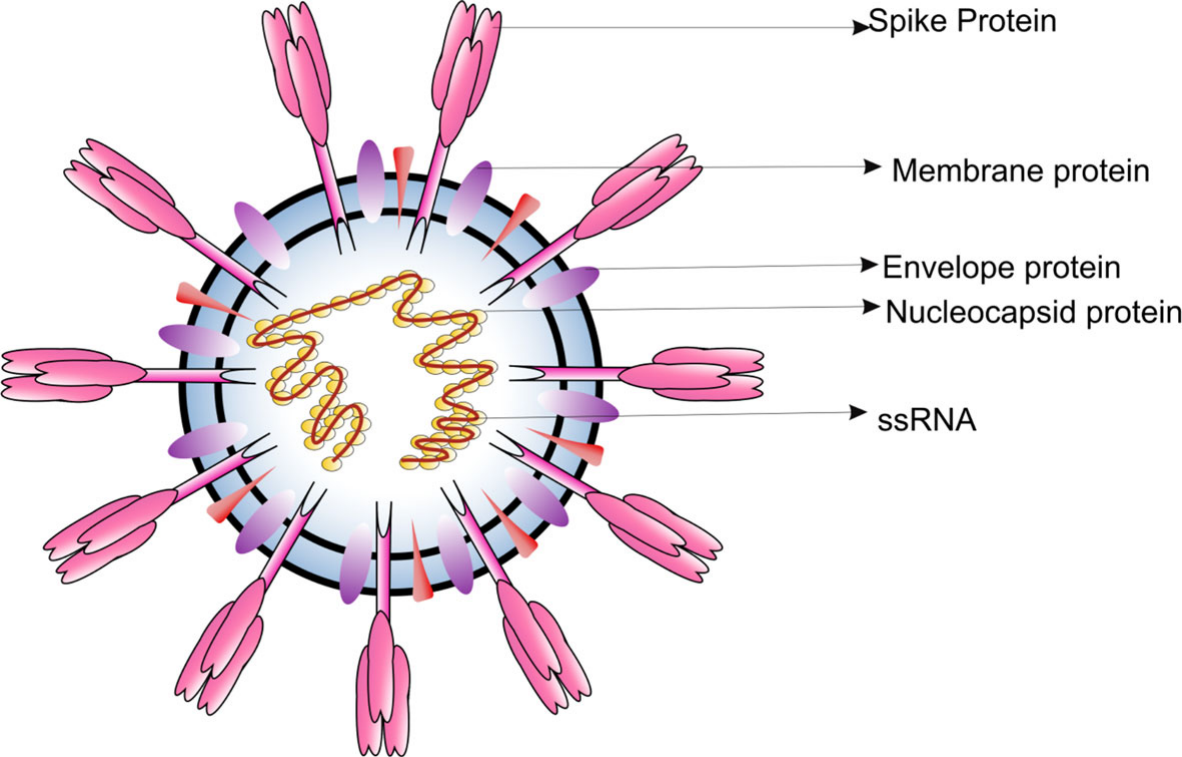
Diagrammatic representation of the structure of respiratory syndrome causing human CoV.

CoVs are microscopic (65-125 nm in diameter), enveloped, single stranded positive sense RNA viruses, with a genome size of 26 to 32kb (**Figure 1**). Based on the phylogenetic analyses, the CoVs can be classified into subfamily Orthocoronavirinae, subdivided into four genera; *BetaCoV, DeltaCoV, AlphaCoV and GammaCoV* (1). The *Alpha-* and *BetaCoVs* can infect mammals whereas the *Gamma *and *DeltaCoVs *mostly infect birds (2). Examples of *Alpha- and BetaCoVs* which have severe implications on livestock include porcine enteric diarrhea virus (PEDV), porcine transmissible gastroenteritis virus and swine acute diarrhea syndrome CoV (SADS- CoV). CoVs are also known to induce respiratory burden in dogs [canine respiratory CoV (CRCoV)], progressive demyelinating encephalitis in mice [mouse hepatitis virus (MHV)] and gastrointestinal symptoms in various animals [bovine CoV (BCV), canine CoV (CCoV), feline CoV (FCoV), transmissible gastroenteritis CoV (TGEV), and turkey CoV (TCV)] (3). Interestingly, all CoVs responsible for virally induced pandemic in humans belong to the *BetaCoVs*. Since most of the CoVs are capable of infecting animals it is believed that wild animals are the natural reservoirs of CoVs. However, many CoV species also inhabit commercial and domestic animals.

**Figure 2:**
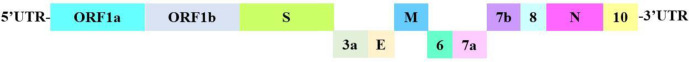
Schematic representation of SARS-CoV-2 genomic sequence. UTR, Untranslated region; ORF, Open reading frame; S, Spike protein; E, Envelope protein; M, Membrane protein; N, Nucleocapsid protein.

The first CoV infectious bronchitis virus (IBV) was discovered in 1930, which caused acute respiratory infection in chicken. Two additional animals CoVs were isolated during 1940 and were characterized as TGEV and MHV (4). In 1960, the first human CoVs, namely human CoV OC43 and human CoV 229E were isolated from humans. SARS-CoV (5, 6), HCoV-NL63 (7)and HCoV-HKU1 were identified in 2004 in China, Netherlands and Hong Kong, respectively (8, 9). Moreover, MERS-CoV (Middle East respiratory syndrome CoV) or MERS-CoV CoVEMC/2012 (MERS CoV Erasmus Medical Center/2012) was first detected in Saudi Arabia (10), and SARS-CoV-2 was reported in 2019 in China (11). Furthermore, a detailed study of SARS-CoV-2 genomic sequence (**Figure 2**) revealed that it is very closely associated with the 2003 pandemic related SARS-CoV (11, 12).

## The Search for an Intermediate Host

In general, domestic animals serve as the intermediate host for transmitting these viruses to humans (13). Notably, all the HCoVs which originated in animals were detected in bats; thus, bats likely represent a major reservoir of *Alpha-* and *BetaCoVs* (13). SARS-CoV-2 is predicted to have a bat origin due to the high sequence homology of RNA dependent RNA polymerase (RdRp) and S (spike) gene with bat CoVs (RaTG13) (14). The presence of an intact single ORF8 in the genome is yet another indicator of its bat origin. Though bats are considered the natural host, no direct progenitor of the virus has thus far been detected, therefore, it is likely that SARS-CoV and SARS-CoV-2 were newly produced viruses as a result of recombination of already existing bats species (15). Cui et al. showed that SARS-CoV progenitors originated as a result of recombination in bat CoVs which eventually infected the palm civets and humans (13). Furthermore, a genomic recombination study stated that bats possessed several CoV subclades which consistently participated in recombination with HCoVs. Thus, the diverse genetic pool presented by bats may have contributed to SARS-CoV-2 origin (16). Additionally, bats, the only flying mammals, have several advantages like the ability to fly long distances, stay in a clustered habitat, longevity, apt body temperatures, etc., which makes them a suitable incubation host for these RNA viruses (17). In the case of SARS-CoV, masked palm civets (*Paguma larvata*) sold widely at Guangdong’s wet markets were investigated to be the intermediate host (18). Variation in the receptor binding domain (RBD) of S gene and open reading frame 8 (ORF8) essential for angiotensin-converting enzyme 2 (ACE2)-mediated interaction of the virus and crucial for transmission of the virus from civets to humans were identified (19, 20).

Dromedary camels serve as an intermediate host for MERS-CoV (21). Importantly, camels from the Middle East, Africa, and Asia had MERS-CoV-specific antibodies (22–24). Also, *Tylonycteris *bat CoV HKU4 and *Pipistrellus *bat CoV HKU5 are phylogenetically related to MERS-CoV (25, 26). Furthermore, the recently identified human infecting SARS-CoV-2 (27) shared greater than 80% and 50% resemblance with SARS-CoV and MERS-CoV, respectively. Intriguingly, the RBD of the S protein is similar in amino acid sequence to that of SARS-CoV, thus implicating that these viruses share a similarity in receptor binding (28). A phylogenetic study revealed that SARS-CoV-2 shared similarities to Malayan pangolin (*Manis javanica*) CoV (pangolin-CoV-2020). The CoV from pangolin is also known to share homology with the bat CoV (29). Thus, pangolins are predicted to be the SARS-CoV-2 intermediate host However, one study supports the possibility of rodents or squirrels acting as possible intermediate hosts (30).

## Impact of SARS-CoV-2 on Human Population

**Figure 3:**
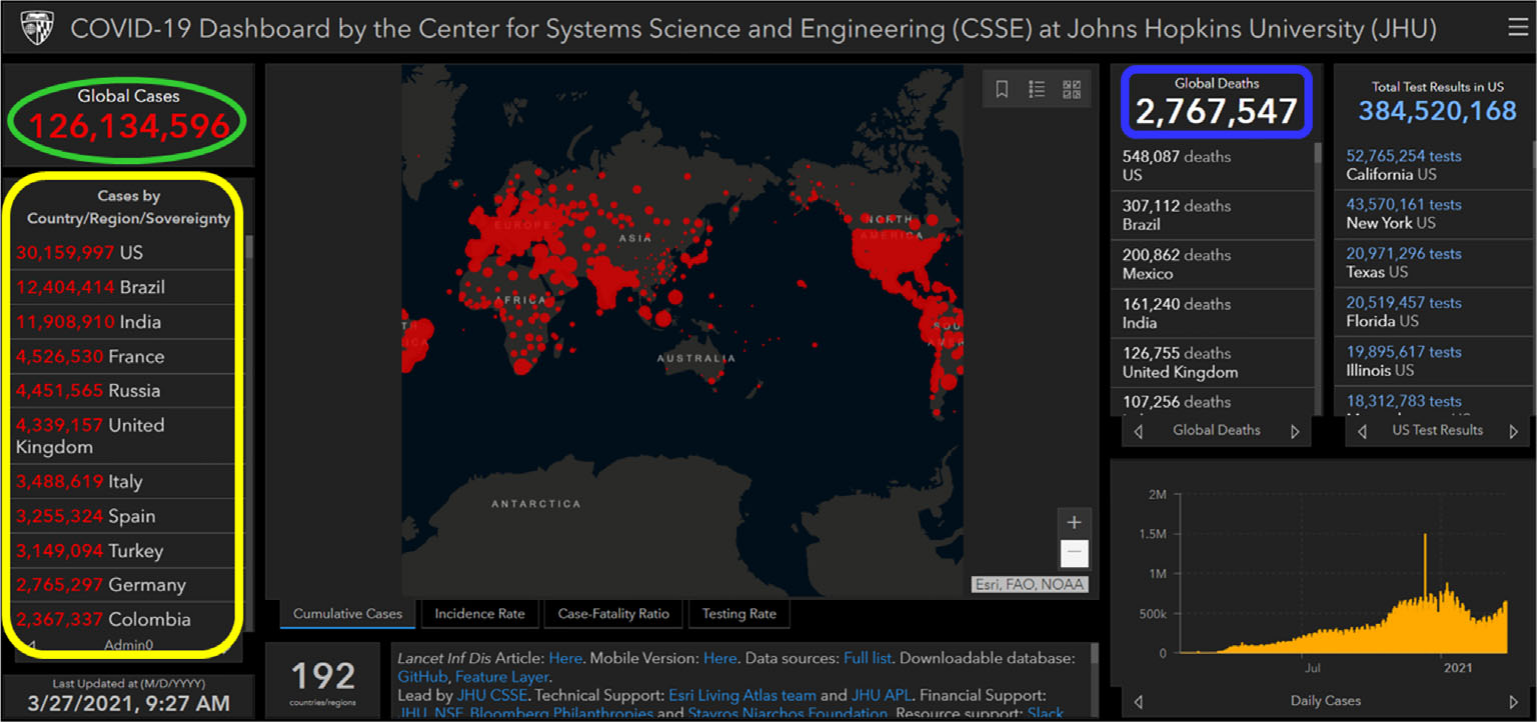
Image showing a screenshot as of 27 March 2021 (9:27 AM) of a collective global map of COVID-19 cases by the Center for Systems Science and Engineering, Johns Hopkins University, United States (37). This database is updated constantly and can be viewed at https://www.arcgis.com/apps/opsdashboard/index.html#/bda7594740fd40299423467b48e9ecf6. On the left-hand side, region/country showing nine most infected COVID-19 cases (circled in yellow color), on the top left-hand side global infected cases are showing (circled in green color), and on the top right-hand side global deaths are showing (circled in blue color).

The initial cases of SARS-CoV-2 infection represented by severe pneumonia appeared in December 2019 (31). Human-to-human transmission of SARS-CoV-2 was first reported and confirmed by a group of clinicians and scientists from the University of Hong Kong (12). CoVs are known to cause respiratory illnesses which may vary from mild to severe manifestations and gastrointestinal tract infections in humans (32). The wide spectrum of COVID-19 extends from benign respiratory distress, self-resolving, to extreme progressive pneumonia, multiple organ failure, and death (33). The first known infected individuals were related to the wet markets of Huanan South China (34). The illness caused by the virus was termed CoV disease of 2019 (COVID-19), and declared a pandemic on 11 March 2020 by the World Health Organization (WHO) (https://www.who.int/dg/speeches/detail/who-director-general-s-opening-remarks-at-the-media-briefing-on-covid-19—11-march-2020). The disease rapidly spread from China to several countries across the globe (35). Since then, several countries have adopted serious measures to provide effective disease prevention and surveillance strategies for their population (12, 36). Based on the CoV Resource Center at Center for Systems Science and Engineering, Johns Hopkins University, United States (US), as of 27 March 2021 (9:27 AM), the total number of confirmed SARS-CoV-2 cases worldwide were 126,134,596 of which 2,767,547 (2.19%) were fatal (https://www.arcgis.com/apps/opsdashboard/index.html#/bda7594740fd40299423467b48e9ecf6). **Figure 3** shows an overview on how severe the infection has spread globally. On the top of the list, US recorded more than 30 million cases, followed by Brazil (> 12 million), India (> 11 million), France (> 4.5 million), Russia (> 4.4 million), United Kingdom (UK) (> 4.3 million), Italy (> 3.4 million), Spain (> 3.2 million), Turkey (> 3.1 million), Germany (> 2.7 million), and Colombia (> 2.3 million).

Elderly individuals, mostly in the age group of 25 and 89 years, especially with a comorbid condition or a compromised immune status, were associated with SARS-CoV-2 infection (38). The cases of COVID-19 were least among infants and children (38). In addition, studies reported the susceptibility of male individuals to COVID-19; however still controversies exist (39). The epidemiological features and clinical manifestations resulting from COVID-19 are poorly characterized, as they are primarily dependent on case reports from vivid regions.

## Impact of Environmental Factors on SARS-CoV-2 Transmission

**Figure 4:**
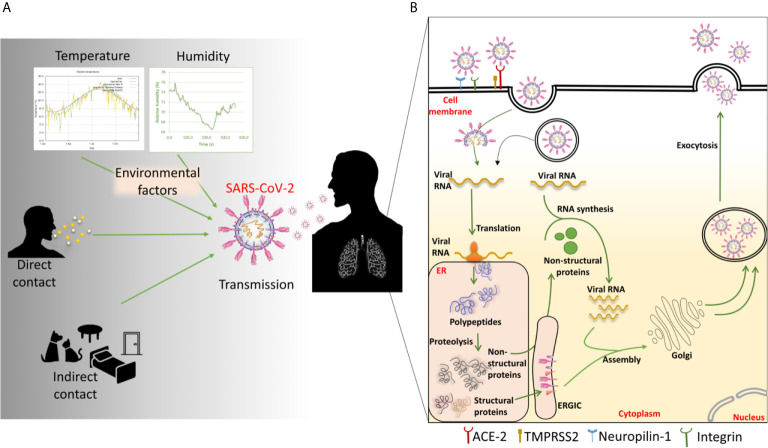
**(A)** Factors involved in SARS-CoV-2 transmission and **(B)** mechanism of SARS-CoV-2 entry into the host cell. The transmission of SARS-CoV-2 occurs through various direct and indirect routes. Environmental factors like temperature and humidity show the impact on the transmission. Once the virus enters inside the host, it enters into the cell through angiotensin-converting enzyme 2 (ACE-2). It can also use various other cell surface receptors like neuropilin-1 to facilitate the entry and infectivity. By the membrane fusion or endosomal pathway, the virus releases its genetic material inside the cytoplasm. The viral RNA then gets transcribed and translated into polypeptides. The polypeptides are cleaved to generate various structural and non-structural proteins (nsps). The nsps help in viral replication and transcription. The more viral RNA copies are produced through the replication. Meanwhile, the structural proteins like E, M, and S proteins get embedded in the endoplasmic reticulum (ER) membrane and transported to Golgi through ER-Golgi intermediate complex (ERGIC). The multiple numbers of new virions are then assembled and exocytosed of the cell to infect nearby cells.

SARS-CoV-2 can be transmitted by being in direct contact with an infected individual or indirect contact through virus-contaminated premises or surfaces (40, 41) (**Figure 4A**). Domestic animals live close to the human environment and can act as carriers of a variety of pathogens (44). Recent reports have demonstrated that SARS-CoV-2 has potential to infect cats, ferrets, Syrian hamsters and dogs (43–46). The cats are susceptible to airborne transmission. These animals may act as carriers and source of transmission of the disease within the animal or human population.

Climatic factors play an essential role in the spread and evolution of any pathogen and disease. Like influenza viruses, CoVs display a seasonal infectivity pattern, most prevalent from December to April (47). In particular, 229E and OC43 are responsible for 15–29% of the overall cases of common colds (3).

A study reported time series analysis for the impact of temperature and humidity on COVID-19 transmission focused on the area of Hubei province from where the pandemic originated (48). A temperature rises of 1°C daily and humidity ranging from 67% to 86% caused reduction in cases by 36-57%. Additionally, when the temperature was between 5-8.2°C and relative humidity was increased by 1% daily, confirmed cases were reduced by 11% to 22%. Thus, an increase in temperature and humidity was correlated to lower levels of COVID-19. Another study with cases from China and USA, also stated that higher temperature and humidity was associated with lower transmission rates (49). The study also depicted that, post lockdown in the included countries, temperature and humidity was still affecting the transmission but with lesser intensity. Contrary to this, another study from China does not support the hypothesis that high temperature or UV radiation can reduce COVID-19 transmission (50).

Another investigation, for 166 countries (excluding China) suffering from the COVID-19 outbreak, by Wu et al. (51), concluded that temperature and humidity are inversely proportional to daily new COVID-19 cases and deaths. A 1°C rise in temperature reduced the appearance of new cases and deaths by 3.1% and 1.2% respectively. While 1% rise in relative humidity reduced new cases and deaths by 0.9% and 0.51% respectively. A temperature, humidity and latitude analysis study were performed by Sajdi et al to predict COVID-19 spread and seasonality (52). It depicted that meteorological status was stable prior to the month of disease outbreak and hence aided in the droplet stabilization and enhanced viral propagation. The spread of COVID-19 was significant in the 30-50° N’ longitude corridor with similar weather patterns of temperature 5-11°C, and low specific (3-6 g/kg) and absolute humidity (4-7 g/m^3^).

A study performed by Juni et al, capturing the initial period of the pandemic in March 2020, focused on the effect of social factors like school closures, restrictions of mass gatherings, measures of social distancing and geographical factors like latitude, temperature and humidity on COVID-19 transmission (53). Restrictions in mass gatherings, school closures and measures of social distancing led to a 35%, 37%, 38% relative reduction in epidemic growth respectively. While there was 9% relative reduction in epidemic growth when relative humidity was increased by 10% and 8% relative decrease was observed with increased absolute humidity per 5 g/m^3^. In this study, there was no correlation found either with temperature or latitude. A report conducted on EU-15 countries also states that 1°C rise in temperature slightly reduced the transmission rate (54). A few other reports have also demonstrated that there is a negative correlation in temperature and COVID-19 transmission and/or associated deaths (55–57).

In the case of African regions, Meo et al. conducted a study to analyze implications of weather conditions on incidence and mortality of COVID-19 pandemic in 16 highly populated countries. The output of the study suggested an increase in relative humidity and temperature decreases rise in new COVID-19 cases and death (58). Another study focusing 52 African states also suggested a similar scheme as Meo et al., 2020, that the mean temperature and relative humidity are inversely related to COVID-19 growth in African region (59). As per rapid expert consultation provided by National Academies of Science, Medicine, Engineering, which reviewed some published and unpublished studies, decreased transmission was seen in excessive ambient temperatures and humidity (National Academies of Sciences, Engineering, and Medicine 2020). However, there are various limitations to the studies conducted so far. It has been 16 months since the pandemic has started and the studies conducted so far considers only a short. Hence, it is difficult to assess the long-term trend accounting for the different seasons for particular areas under study. Also, different countries implemented various strategies to control the COVID-19 transmission which actually could have impacted the reduction or increase in cases instead of meteorological reasons (60). Conclusively, based on current evidence, the higher temperature, relative humidity and absolute humidity were associated with fewer COVID-19 cases. However, more studies are required in the future to decode the relation of temperature, humidity and various other environmental factors in worldwide COVID-19 transmission.

## Mutation Rate of SARS-CoV-2

SARS-CoV-2 has a high transmission rate within the human population throughout the globe. In general, CoVs undergo modification at a rate of 10^4^ substitutions in a year per site. The mutation rates for SARS-CoV and MERS-CoV whole genome is assessed to be 0.80 – 2.38 × 10^3^ and 1.12×10^3^ nucleotide substitutions per year at a site (61, 62). HCoVs recognize different host receptors to enter the hosts; for example, HCoV-OC43 and HCoV-HKU1 recognize various O-acetylated sialic acid moieties (63), HCoV-229E interact through the human aminopeptidase N (hAPN), MERS-CoV uses dipeptidyl peptidase-4 (DPP4) (64), and HCoV-NL63 and SARS-CoV binds to the ACE2 receptor (65, 66). ACE2 and Transmembrane Serine Protease 2 (TMPRSS2) on the host cells function as entry receptors for SARS-CoV-2 (67). Noticeable mutations in SARS-CoV-2, which help the virus distinguish from the rest of the *beta CoVs*, include multiple mutations in the RBD of the S protein, a furin-like protease site at the S1/S2 interface, and the presence of 3 O-linked glycans encompassing the protease site (68–70).

It is predicted that SARS-CoV-2 evolves within the host after infection, thereby modulating its transmissibility and infectivity potential (71). Recent research studies showed that the new SARS-CoV-2 has evolved with many mutations and developed the ability to cross species. One such variant is the SARS-CoV-2 VUI 202012/01 (Variant under Investigation, year 2020, month 12, and variant 01) detected in the United Kingdom. The variant is known to have 14 mutations and three deletions in its sequence. In particular, two of the identified mutations, N501Y and P681H, result in altered RBD and are predicted to influence virus transmissibility (https://www.who.int/csr/don/21-december-2020-sars-cov2-variant-united-kingdom/en/). Mutations at Tyr442, Leu472, Asn479, Asp480, Thr487, and Tyr491 of SARS-CoV S-protein Coveney are considered crucial for its function (72). Notably, the RBD of SARS-CoV-2 differed at 5/6 residues compared to SARS-CoV, which may have promoted the binding of the virus to host ACE2 (73). Specific mutations in the RBD of SARS-CoV-2 enabled the virus to evade the neutralizing antibody response. D614G mutation was predominant in most strains of the SARS-CoV-2 and is predicted to have influenced the spread of the virus (74, 75). This mutation first identified in China and Germany is widely spread in various areas of Europe. Besides, variation, i.e., a 382-nucleotide deletion, in the virus ORF8 resulted in milder infection (76). A study involving glycan profiling investigation indicated that the SARS-CoV-2 S protein is known to possess heterogenous N-linked glycans. Finally, glycan profiling and a detailed understanding of virus evolution are necessary to develop an effective drug and vaccine strategy.

## SARS-CoV-2 Variants

Recently, several new SARS-CoV-2 variants have been identified resulting in clusters of outbreaks and, in some cases, global dissemination. Any of these variants contains a range of mutations, some of which are essential to the viral genome’s function. The majority of these mutations are detrimental to the virus and hence disappear by natural selection. However, some SARS-CoV-2 variants can be more transmissible, may affect its ability to be identified by diagnostic tests, have resistance to therapeutic agents, may evade natural and vaccine-induced immunity, and may have an effect on COVID-19 morbidity and mortality (77). This viral evolution is a well-characterized phenomenon observed in seasonal coronaviruses (78–80) and has been recently replicated *in vitro* (81).

Viral mutations and variants are regularly monitored by sequence-based analysis, experimental studies, and epidemiological data. They are categorized generally as variants of interest, variants of concern, and variants of significance. The SARS-CoV-2 variants of concern spread at least 20% to 50% more easily from person to person. This enables them to infect a greater number of individuals and evolve more rapidly and extensively, gradually establishing themselves as the dominant strain.

Recently, the initial optimism about the development of COVID-19 vaccines has been dashed by the appearance of new SARS-CoV-2 variants (82). Current coronavirus vaccines were developed for older versions of the virus, but scientists say they the immune response could be weaker and less long-lasting. Currently there are five variants of concern:

*The B.1.1.7, lineage*, originated in the U.K, it soon spread to other countries after its discovery in December 2020 and grew at an exponential pace. B.1.1.7 tends to be more contagious due to many mutations in the coronavirus’s spike protein. Mutations include N501Y, which makes the virus latch on to human cells more closely, but is unlikely to aid the virus in evading existing vaccinations; P681H, which may aid infected cells in the production of new spike proteins more efficiently; and the deletions H69–V70 and Y144/145, which change the form of the spike and can help evade certain antibodies (83).

*The B.1.351 Lineage*, first reported in South Africa in December 2020, this variant, also known as 20H/501Y.V2 carries a mutation (N501Y) in the spike protein’s RBD, which lead to increased transmission, with estimates ranging between 40% and 70%; K417N, which also aids the virus in binding to human cells more closely; and E484K, a mutation that may aid the virus in evading certain types of antibodies (84).

After spreading from South Africa into neighboring countries, it has expanded to at least 68 nations. Scientists are worried about the variant because clinical trials have shown that individuals who recover from other variants may not be protected against B.1.351 (84).

*The P.1 lineage* was discovered in four people in Japan who contracted P.1 while on vacation in Brazil. The lineage first appeared in late 2020 in Manaus, Brazil’s largest city in the Amazon region. It quickly became the dominant variant in that city, and it has now spread to at least 37 countries.

P.1 is a close relative of the B.1.351 lineage, and it shares some of the same coronavirus spike protein mutations. The spike protein’s critical mutations are identical to those seen in the B.1.351 lineage, but may have occurred independently: N501Y and K417T, which could facilitate the virus adherence to its host; and E484K, a mutation that may aid the virus in evading certain types of antibodies (85).

*The CAL.20C variant* was originated in California, in late 2020, from the B.1.427 and B.1.429 lineages. This variant shows the L452R mutation and may give the coronavirus an advantage in spreading over other variants, but the results of experiments to prove this have yet to be published. About 45% of existing samples in California contain this mutation. Two reports indicate that CAL.20C is more infectious than older strains of the coronavirus, but it does not seem to be circulating as rapidly as variants such as B.1.1.7 (86).

*The B.1.617 variant*, also known as G/452.V3, was first detected in Maharashtra, India, in October 2020. This variant has been described as a super-spreader, prompting the closure of five cities (Lautoka, Nadi, Suva, Lami, and Nausori), accounting for nearly two-thirds of the country’s population. The variant was first observed in other countries in late February 2021, including the United Kingdom on 22 February, the United States on 23 February, and Singapore on 26 February 2021. The B.1.617 variant shows the E484Q and L452R mutations, which confers a higher binding capacity to the human ACE2 receptor, as well as a greater ability to escape host immune systems. Recent studies shows that B.1.617.2 is at least as transmissible as the B.1.1.7 variant. A research conducted by the Centre for Cellular and Molecular Biology (CCMB), in Hyderabad, discovered that sera from Covishield-vaccinated people protected against the B.1.617 variant (87).

Further research is required about the protection against infection and disease provided by the current generation of SARS-CoV-2 vaccines in respect of existing and possibly evolving viral variants of concern. Additional research is required on the role of T cell-mediated immunity against SARS-CoV-2 and its role in viral variant selection. Another critical concern is whether updated vaccines can be developed and implemented relatively soon (82).

## SARS-CoV-2 Interaction With Host

The overall lifecycle of all the CoVs shows a similar pattern of viral entry inside the cell till the viral egress from the infected cell (**Figure 4B**). The life cycle of the CoV begins upon (i) interaction of its S protein with the host receptor, followed by (ii) virus entry into the host cell, (iii) intracellular viral replication and transcription, (iv) protein production, assembly, and release of new virions (88). The virus can attack the epithelial lining of the exposed part of a person who has encountered the virus through any of the transmission routes. After entering into the cells, the virus multiplies and produces several copies for further spread inside the body. SARS-CoV-2 utilizes the ACE2, similar to SARS-CoV, to gain entry inside the cells. ACE2 is expressed by a variety of body cells including the lung epithelia where SARS-CoV-2 predominantly attacks. However, recent reports suggest various other targets where SARS-CoV-2 can possibly attack (38, 89, 90). The various aspects regarding SARS-CoV-2 entry are still under study. Recently, Neuropilin-1 has been shown to facilitate SARS-CoV-2 entry and infectivity (91). Some other reports propose that integrin can also aid in the entry of SARS-CoV-2 inside the cells (92, 93). The involvement of neuropilin and integrins in SARS-CoV-2 entry may strengthen the virus to enter in various cells where these receptors are prominently expressed as compared to ACE-2. Additionally, various signaling pathways other than those involved in ACE-2 mediated entry can also get triggered leading to changes in canonical cellular response (94).

SARS-CoV-2 utilizes a three-step method for its fusion with the cell membrane, which involves receptor binding followed by the change in S protein conformation and subsequent cathepsin proteolysis by cytoplasmic proteases and induction of membrane fusion in endosome (95–97). Further, the endosome opens and the virus is released. The host proteases which generally hydrolyze the endogenous protein, can facilitate in the degradation of viral N protein leading to viral RNA uncoating (98). Another suggested mechanism for entry into the cytoplasm is that the S protein binds to ACE2 and then cleaved proteolytically by host serine protease TMPRSS2 (99, 100). Interestingly the host-pathogen membrane fusions occur at low pH *via* S2 subunit of S protein. SARS-CoV-2 possess a site for furin-mediated cleavage which is absent in SARS-CoV (67). Recently it has been revealed that inhibitor of a serine protease and cathepsin halts entry and growth of SARS-CoV-2 in human airway epithelial cells *in vitro* (67). By either way, the viral genomic RNA is released into the infected cell cytoplasm. The replication and transcription of the viral genome are carried out by different non-structural proteins (nsps) encoded by the viral genome. The generated positive RNA genome is translated to replicases from ORF1a/b of the virus. This then works to produce more RNA viral genome copies. The viral structural proteins, translated from positive RNA strands, like E, M, S proteins get integrated to the endoplasmic reticulum membrane and continue to the endoplasmic reticulum-Golgi intermediate compartment (ERGIC) (101, 102). The viral genome RNA coated with N proteins then gets encircled by vesicles from the ERGIC compartment to form virions. These are further exocytosed out of the cell to infect nearby cells and establish the infection in the microenvironment (103).

## Host Immune Response to SARS-CoV-2

When the virus enters the host cells and starts propagating inside the system, the host immune system tries to tackle the infection in all possible ways. The host system uses swords and shields of cellular machinery involving immune cells and molecular machinery like cytokines, chemokines including various cell signaling factors against the invader. During or post viral entry, the virus may get recognized by host cells using pattern recognition receptors (PRRs). Further, the cellular defense system secrets a variety of signaling molecules to invite immune cells at the site of infection. The immune cells process the viral antigens post endocytosis and are then processed inside these cells. Additionally, the processed viral peptides are displayed to the cell surface through major histocompatibility complex (MHC) class I proteins and presented to cytotoxic T lymphocytes (CD8+). These further become activated, demonstrating clonal expansion leading to virus-specific effector and memory T lymphocytes. These T lymphocytes lyse the virus-infected tissue cells. Also, by another way, the virus particles or peptides can be processed by antigen-presenting cells for displaying through MHC II molecules to CD4+ T lymphocytes. The CD4+ cells get activated to release cytokines and drive CD8+ T cell expansion. Further, the CD8+ T cell can kill other infected host cells. On the other hand, the B cells can also interact with CD4+ T lymphocytes or can recognize the virus directly. Post interaction, the cytokines released from with CD4+ T cells accelerate the B cell activation leading to clonal expansion and specific antibody production to neutralize the virus and ultimately control the spread of infection.

Post viral entry inside the cell, the major PRR involved in recognizing the viral material that is pathogen-associated molecular patterns (PAMPs) are RIG-I, MDA-5, TLRs (TLR3 and TLR7) which recognizes cytosolic RNAs. These PRRs further drive immune molecule signaling. *In vitro* studies have shown that SARS-CoV-2 induces RIG-I pathway (104). Another pathway induced by SARS-CoV-2 inside cells includes NOD-like receptor (NLR) activation and tumor necrosis factor (TNF)-α production (104). The inborn genetic error in TLR3 signaling pathways result in altered type I interferon (IFN) response and are shown to be associated with lethal COVID-19 (105). IFNs are considered as primary antiviral cytokines. In the case of SARS-CoV-2 infection a decreased IFN responses has been observed *in vitro* and in animal model (106). In SARS-CoV-2 infected human lung tissues as well, the IFNs were not significantly induced (107). The low secretion of IFN post SARS-CoV-2 is correlated to immune evasion strategy of the virus (108, 109). Recent studies have shown that the SARS-CoV-2 proteins like Nsp13 (a helicase– triphosphatase), Nsp15 (an endonuclease), and ORF-9b targets IFN pathway by interacting with TBK1, RNF41 and TOMM70 respectively (110, 111).

The subsequent immune activation post infection makes alteration in the balance of acute phase reactants in the peripheral system. In case of SARS-CoV-2 infection, higher levels of D-dimer, c-reactive protein (CRP), and procalcitonin (PCT) were associated with severe disease compared to non-severe disease (105). Various reports have observed a correlation between COVID-19 severity and the elevated levels of IL-6, alanine aminotransferase (ALT), lactate dehydrogenase (LDH), high-sensitivity cardiac troponin I, creatinine, creatine kinase, D-dimer, serum ferritin, prothrombin time (PT), CRP etc. (36, 112). Once SARS-CoV-2 establishes its infection in the lung microenvironment, COVID-19 development is accelerated due to increased load of viral infection as well as host response produced against the infection. Just like SARS and MERS, the severe COVID-19 cases have shown a common manifestation of cytokine storms (113, 114) Particularly, COVID-19 severity and cytokine syndrome observed in the disease is clinically associated with older patients i.e., above the age of 65 yes compared to those under 65 (115, 116). It’s uncertain why older people are more vulnerable to cytokine storms, although one explanation is that it’s due to NLRP3 activation which is an inflammasome component. During aging, the gradual elevation in abundance and activity of NLPR3 is observed in immune cells including alveolar macrophages. Age dependent increase of NLPR3 in alveolar macrophages have been shown to contribute in pulmonary fibrosis (115–117). In a two-phase model suggested by Mueller et al, NLRP3 basal overactivation may be the first step, followed by SARS-CoV-2 antigen-mediated hyperactivation, which causes the cytokine storm. Also, in elderly people, due to age progression, the capacity of immune response declines leading to reduced protection against pathogens, this is referred as immunosenescence (118). During onset of infection, immunosenescence drives more active innate immune response leading to chronic and low-grade inflammation (118). This may further be correlated to increased cytokine storm in elderly patients and subsequent COVID-19 severity. Post-infection driven inflammasome activation in immune cells, epithelial cells and endothelial cells release cytokines, interleukin (IL)-1β and IL-18 which is proportional to the severity of COVID-19 symptoms (100). The toll-like receptors (TLR) displayed on the host cells acts as viral RNA sensing machinery and induce NF-kB pathway which activate a plethora of pro-inflammatory cytokines leading to virus-induced inflammation (119).

Reports suggest that SARS-CoV-2 viral protein targets several innate-immune signaling proteins (110, 111). As stated earlier in this section, Nsp13, Nsp15, and ORF-9b targets IFN pathway. The Nsp13 also interacts with TLE1, TLE3 and TLE5 while the Orf9c interacts with NLRX1, F2RL1 and NDFIP2, which are involved in NF-κB pathway. ORF6 may hinder IFN signaling by targeting NUP98–RAE1 (110, 111). A study exploring transcriptional changes in immune cells and samples of bronchoalveolar washing of COVID-19 patients analyzed the different cytokine expression modulations. In a bronchoalveolar lavage fluid (BALF) sample the expression of CCL2, CCL8, CCL3L1, CXCL2, CXCL1, IL33 was upregulated. While in peripheral blood mononuclear cells (PBMC) CXCL-10, TNFSF10, TIMP1, C5, IL18, amphiregulin, neuregulin1, and IL10 were found to be induced post-COVID-19, which denotes the cytokine storm in patients (120). The overall exaggerated immune response, subsequent cytokine storm, the viral infection leads to the development of acute respiratory distress syndrome in COVID-19 patients. Various comorbidities associated with COVID-19 add up in severity of the disease can be correlated to mortality in many cases (121).

To control spread of infection inside the body, the adaptive immune response plays a crucial role. Post the first week of symptoms onset, virus-specific antibodies of IgM isotype are observed in the patients. Subsequently, IgG response is also observed that may retain long term immune memory (122). IgM and IgG responses against N protein and RBD of S protein were observed in non-severe as well as severe patients in the middle age group (with IQR, 34–56 years) after 10 days of first symptom generation (122). Wajnberg et al. studied more than 30,000 COVID-19 patients with mild to moderate symptoms and concluded that the neutralizing antibodies persisted at least 5 months (123). Some other reports also state that these antibodies may persist up to four months while other reports have presented the alleviation of neutralizing antibodies before four months itself in COVID-19 patients (post 4-12 weeks of symptoms onset) (124). A recent report analyzing neutralizing antibody response dynamics in patients who have recovered from COVID-19 states that it may vary greatly, and prediction of immune longevity can only be accurately determined at the individual level (125). Various geographical regions and age groups are necessary to understand more in this context.

There exists heterogeneity in CD4+ and CD8+ T cell response in non-severe and severe COVID-19 (126–128). Some studies have shown decreased cytokine response while others report aggressive CD8+ response with high cytotoxic effect (126). An increased CD8+ response against the proteins of SARS-CoV-2 was observed in patients with mild COVID-19 as compared to severe cases, thus indicating a protective role against the virus (129). The CD4+ cells showed impaired function as well as alleviated IFN-γ secretion in severe COVID-19 patients with higher age and comorbidity index (127). Grifoni et al. conducted a study to analyze T cell response against SARS-CoV-2 proteins and focused on non-hospitalized, recovered COVID-19 patients after 20-35 days of symptom onset (130). The report demonstrated the presence of virus specific CD4+ cells in 100% and CD8+ cells in 70% of recovered COVID-19 patients (130). This indicates virus specific T cell memory. However, the efficacy of these cells to protect from re-infection is not explored further. Interestingly, the T cell responses were against internal viral proteins instead of surface glycoprotein (130). In another report considering mild as well as severe COVID-19 patients post recovery, compared to other viral proteins the CD4+ response was more against spike protein of SARS-CoV-2 (129). SARS-CoV-2-reactive CD4+ T cells were also detected in ∼40%–60% of unexposed individuals (130). This hints towards the cross-reactive T cell recognition between circulating “common cold” coronaviruses and SARS-CoV-2 (130).

There are some other important subtypes of CD4+ T cells which include regulatory T cells (T-reg cells) and Th-17 cells. T-reg cells play a crucial role in maintaining overactive immune response while Th-17 cells promotes inflammatory cytokine production (131). The balance between these two plays important role in response to infection. In severe COVID-19 patients, increased Th17 cytokines while decreased T-reg numbers has been observed (131). Moreover, in these patients the increased IL-6 could promote Th-17 activation and T-reg inhibition (131). Such a mechanism during COVID-19 favors imbalance of Th-17 and T-reg cells ratio and may aid up in severity (131). Further exploration is yet required to decode precise roles of CD4+ and CD8+ T cells against acute SARS-CoV-2 infection as well as in protection from future infection.

## Therapeutics Strategies

Till date, no potential therapeutic agents (antivirals) have been discovered to treat specifically this global disease. Currently, researchers all over the world are working aggressively to discover a potential drug. Drug discovery and development is a tedious/challenging task, takes several years, and involves billions of dollars. Numerous experiments and techniques have been aimed at preventing further spread of COVID-19 and to improve successful and secure therapeutics. Depending on the target, possible anti-CoV therapies can be classified into two categories: (1) those acting on the CoV itself and (2) those acting on the human immune system or cells (132). The production of therapeutic methods is accelerated by elucidating immune responses caused by SARS-CoV-2 (133).

Various pharmacological interventions have been implemented by countries to cure the disease, including currently proven antivirals, various oxygen therapy types, or mechanical ventilation. COVID-19 pandemic demands a rapid development of successful and efficacious therapeutics/pharmaceuticals that can be achieved through application of three principles: (i) the first approach includes investigating and testing the therapeutic efficacy of currently available antivirals (134–136). (ii) Molecular databases and repositories/libraries that can aid in high processing capacity and continuous evaluation of millions of potential therapeutics (135, 137). (iii) Targeted treatment designed to interrupt the virus signaling (cellular and molecular pathways), and immunological response (135, 138–141). The safest and easiest way to produce pharmaceutical products for treatment of SARS-CoV-2 is the first strategy, to find potential molecules from manufactured medicines. It may be accepted by the Green Channel or approved by the hospital ethics committee for accelerated clinical care of patients once effectiveness has been assessed (142). The approaches to drug repurposing can be performed by *in vitro* and *in vivo* studies using a conventional approach, or it can be achieved by a statistical approach, which relies on bioinformatics methods, ‘Big Data’ and ‘Artificial Intelligence’ (AI) to find new indications for drugs already in use, and subsequent validation *in vitro* and *in vivo* (143).

Broad-spectrum antivirals, such as IFNs, ribavirin, and cyclophilin inhibitors have been used to treat CoV pneumonia. The benefit of these therapies is that, since they have been approved for the treatment of other viral infections, their metabolic properties, dosages used, possible effectiveness and side effects are known. However, because these therapies are broad-spectrum, they are unable to directly kill CoVs, and potential side effects should be considered (144). Unfortunately, due to differences in pharmacokinetic (PK) and pharmacodynamic (PD) properties, *in vitro* analysis seldom translates to *in vivo*, posing a significant barrier to clinical translation of a therapeutic candidate (145). Already approved pharmaceuticals, including antimalarials, antiviral, lysosomotropic drugs; and then selected broad spectrum antibiotics, and immunotherapeutic drugs, are the key classes of therapeutic agents that seem effective in COVID-19 therapy and are currently being recommended in various countries (146–154).

Ribavirin is used in the management of many viral infections, including chronic hepatitis C virus, viral hemorrhagic fever, and respiratory syncytial virus, in conjunction with other antiviral medications and, in certain instances, IFN-α. Ribavirin was first commercialized in the early 1980s for the treatment of respiratory syncytial virus in infants, making it the most well-known and widely used antiviral agent. Aside from being regarded as a broad-spectrum antiviral agent against DNA and RNA, which can obstruct viral messenger RNA binding RNA-dependent RNA polymerase production (RpRd), it is also a prodrug that metabolizes into nucleoside analogues that block viral RNA and capping viral mRNA. The combination of nitazoxanide, ribavirin, and ivermectin, as well as zinc supplementation, removed SARS-CoV-2 from the nasopharynx faster than symptomatic treatment (155).

Remdesivir is one of the most effective antiviral medicines for managing coronavirus infection that has been studied. It is an adenosine nucleotide phosphoramidite prodrug with broad-spectrum *in-vitro* antiviral activity against a variety of RNA viruses. Remdesivir, while being a nucleotide precursor, has been shown to prevent viral RNA replication and to prematurely terminate viral RNA transcription by targeting viral RNA-dependent RNA polymerase. For individuals at high risk of hyperinflammation who are diagnosed early during illness (≤10 days) and require supplemental oxygen, the Remdesivir therapy reduced the period to rehabilitation by four days, which is significant improvement for patients and healthcare systems. However, for patients with mild or moderately severe COVID-19 and no need for respiratory support, Remdesivir does not offer significant benefit at day 28. Therefore, Remdesivir is considered as an important COVID-19 treatment option only in selected patient (76).

Ivermectin is an anti-parasitic broad-spectrum drug that has been shown to prevent HIV-1 replication. *In-vitro* analysis performed in Australia, Ivermectin’s antiviral activity on SARS-CoV-2 infected cells showed a 93 percent reduction in viral RNA present in the supernatant and a 99.80 percent reduction in cell-associated viral RNA. A randomized, double-blind, placebo-controlled study of adult SARS-CoV-2 patients revealed that adult patients with mild COVID-19 treated with 12 mg of Ivermectin resulted in an earlier clearance of the virus with a 5-day course. The treatment was found to be both safe and successful. However, to validate these preliminary results, larger studies would be needed (156).

Interestingly, plant based natural products such as plant metabolites are also extensively in use to fight CoV infection (157). Furthermore, various research groups revealed that lipid metabolism (158, 159), RNA-dependent RNA polymerase (16), many virus structural proteins can act as potential drug targets (160) for the management of COVID-19. However, clinical trials are undergoing to screen and translate a potential therapeutic candidate for the management of COVID-19 (132).

The technique of high-throughput screening, which for instance, contributed to the discovery of the anti-HIV infection drug lopinavir/ritonavir, may identify many drug molecules. Through a strategy focused on genomic knowledge and pathological characteristics of various CoVs, the drugs detected would show stronger anti-CoV effects, but the new drug discovery phase could take many years or even more than 10 years to complete (161).

### Therapies That Have the Potential to Act on CoVs

**Figure 5:**
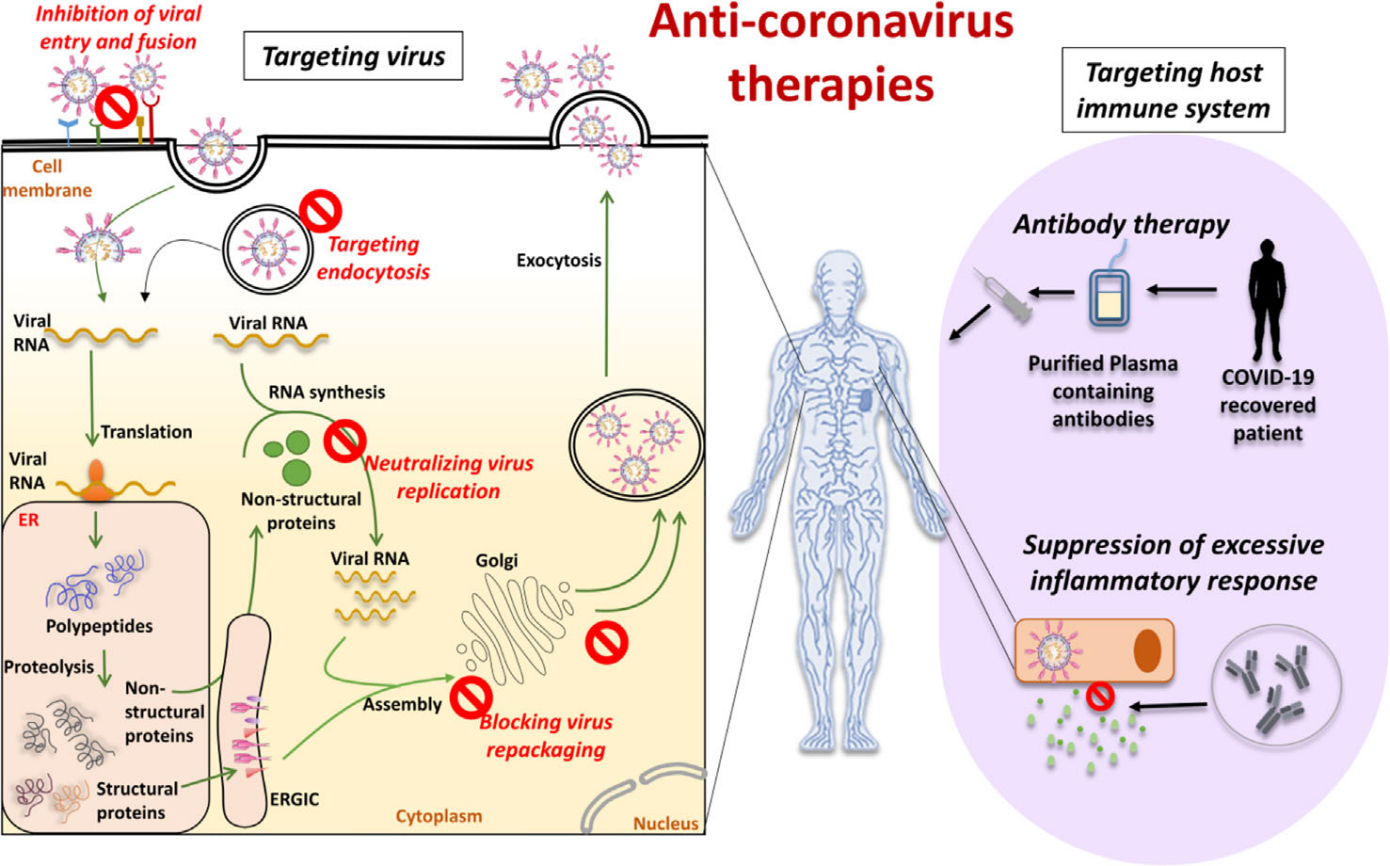
Anti-coronavirus therapies can be based on targeting the virus or targeting the host immune system. Targeting the virus comprises inhibition of viral entry and fusion, targeting the endocytosis, neutralizing virus replication and blocking virus repackaging. Targeting host immune system comprises antibody therapy with plasma from recovered or convalescent patients and suppression of the excessive inflammatory response.

The therapies that focus on the CoV itself include preventing the synthesis of viral RNA by acting on the virus’s genetic material, inhibiting virus replication by acting on essential virus enzymes, and blocking the virus by acting on certain structural proteins that bind to human cell receptors or inhibiting the self-assembly mechanism of the virus. These drugs may be able to engage host receptors or proteases used for viral entry or may affect the mechanism of endocytosis (132). Blocking the signal pathways necessary for human cell virus replication can also have an anti-viral effect. For example, innate immune system response plays an important role in regulating CoV replication and infection. In addition, viruses also bind to cell surface receptor proteins to reach human cells, such as the S protein binding to the receptor ACE2. The therapies that have the ability to work on CoVs can be divided into five broad categories, based on the life-cycle phases of SARS-CoV-2 (**Figure 5**).

#### Inhibition of Virus Entry and Internalization

Some chemical compounds or monoclonal antibodies may block the virus entry or effectively engage the cell surface receptor of the host. To enter into the host cells, SARS-CoV-2 utilizes the S protein present on the viral surface. The protein-protein interaction that exists between the S protein subunits and the ACE-2 receptor’s active site can be targeted to determine an appropriate treatment strategy (162). In particular, the S protein’s RBD is a crucial target for antibody-mediated binding disruption. Many monoclonal antibodies are in preclinical stages of development that show the potential to interrupt this binding (72, 163). Another solution is to provide an excess of the soluble ACE2 that could help to neutralize the virus by competitively binding to SARS-CoV-2 (164). For example, the treatment with recombinant human ACE-2 is under a pilot clinical trial (165, 166). The transmembrane serine protease-2, TMPRSS2, also plays an important role in promoting the entry of the virus into host cells by proteolytically cleaving and activating viral envelope glycoproteins. Mesylate, a clinically proven chemical inhibitor of TMPRSS2, is also capable of substantially reducing infection in cell lines of human lung origin (167).

#### Targeting the Endocytosis of Viruses

Through endocytosis the virus enters into the host cell and release its genetic material for further replication, so the rational goal for antiviral therapy is to block virus-involved endocytosis (168). Clathrin‐dependent endocytosis has been proposed as a potential entry mechanism of SARS‐CoV‐2. AP-2-associated protein kinase 1 (AAK1) controls clathrin-mediated endocytosis. The Janus kinase inhibitor baricitinib was established as a potential candidate drug for SARS-CoV-2 (169). Ouabain, a clathrin-mediated inhibitor, is also being studied for its effectiveness in drug trials for SARS-CoV-2-positive patients (170). Clearly, there would be major therapeutically advantages to avoid entry of SARS‐CoV‐2 at any of these levels.

#### Neutralizing the Virus Multiplication

Some compounds and antibodies could act on enzymes or functional proteins essential to the replication and multiplication of viruses. Multiple drugs against viral proteases, polymerases and helicases have been developed. Clinical studies are currently testing drugs developed against other viral diseases such as Remdesivir (171), Favipiravir, Lopinavir/Ritonavir for their effectiveness in containing the COVID-19 pandemic. Lopinavir and Ritonavir are protease inhibitors targeting SARS-CoV-2 3C-like protease (3CLpro). The key protease of the CoV 3CLpro is responsible for the synthesis of NSP polypeptides. Using high-throughput screening (HTS) for compounds against 3CLpro, prulifloxacin, tegobuvir, bictegravir and nelfinavir have been identified (89, 172). Remdesivir, an antiviral drug developed against Ebola, is an analog of adenosine that is inserted by RNA-dependent polymerases (RdRps) into viral RNA chains and results in premature termination of transcription (171). EIDD-2801 is another oral antiviral drug that, though with lower EC50, functions as a nucleotide analog, such as Remdisivir, and can be administered orally (173).

#### Blocking Virus Repackaging by Targeting Viral Structural Proteins

The proteins of SARS-CoV-2 envelope (E), membrane protein (M) and nucleocapsid protein (N) are essential for the virus survival and propagation and are thus the best drug targets for. Since these viral proteins are somewhat different structurally from the host proteins, there would be minimal adverse effects from drugs targeting these proteins. These structural proteins are also involved in suppressing the host immune system in addition to preserving the viral genome, thus giving the virus a strategic advantage over the host (174, 175). CoV N protein encapsulates viral genomic RNAs during the viral life cycle to protect the genome and co-enter the host cell with viral genomic RNAs, indicating that N is essential for viral RNA replication, particularly at the initiation stage. The N protein works to prevent the silencing of RNA and suppresses siRNA mediated RNA interference. Many siRNA-based therapeutics therefore target the translation of viral E, M, and N proteins and inhibit viral replication, at least *in vitro* (176). The E protein also acts as an ion channel and hexamethylene amiloride inhibits this activity (177). Another PJ34 chemical inhibitor targets the N-terminal domain of N protein in the special ribonucleotide-binding pocket (178). It is important to remember that most of these inhibitors were engineered against the SARS virus; they may not be as effective in combating the current COVID-19 pandemic because of mutations in the SARS-CoV-2 virus. LJ001 and LJ003 are broad-spectrum antiviral compounds that through the production of singlet oxygen molecules, not only inhibit viral entry into the host cells but also damage the viral membrane. Unfortunately, LJ001 is physiologically unstable and is photo-dependent (148). Nonetheless a new class of antiviral compounds is identified by LJ001, and further research into this class of compounds will produce promising results (179).

### Therapies Targeting the Host

#### Suppression of Excessive Inflammatory Response

A well-orchestrated cytokine response is important in the host immune response. Some patients infected with SARS-CoV-2 have been documented to have a hyper-inflammatory response, likely due to a deregulated cytokine response. Compared with non-ICU patients, COVID-19 patients in the ICU were reported to have elevated plasma cytokines, indicating that cytokine dysregulation is involved in the severe type of COVID-19 disease (180). Additionally, as compared to ICU naïve patients, SARS-CoV-2-infected patients admitted to ICU exhibit increased levels of GM-CSF and IL6+CD4+T cells (181). The above facts indicate that inhibition of excessive inflammatory response can decrease the severity of COVID-19 disease. In reducing systemic inflammation, corticosteroids are considered to have excellent pharmacological potential (182, 183). However, their use in patients with COVID-19 is still debatable and needs to be researched in depth. It has been shown that CD4+T cells are stimulated to generate GM-CSF and other inflammatory cytokines following the onset of SARS-CoV-2 infection, resulting in further activation of CD14+CD16+ monocytes with high IL-6 expression (184). This finding leads to the idea that we might theoretically minimize immune stress caused by SARS-CoV-2 by blocking the IL-6 receptor. A multicenter, randomized controlled clinical trial using the IL-6 receptor-specific antibody Tocilizumab is currently underway, in line with this observation (185).

Nanoengineering with potential drugs (such as zidovudine, acyclovir, dapivirine, and efavirenz) opens the door to improved treatment strategies for lung infections. This strategy has the potential to deliver drugs while still providing targeted antiviral action (186). Pulmonary nano-drug delivery systems have special physicochemical properties such as mucosal penetrability, ease of ligand functionalization, improved permeation due to small size, increased local drug concentrations, and robust adjuvant properties for vaccine formulations, rendering them perfect drug delivery systems for the treatment of COVID-19-like pulmonary infections (187).

#### Antibody Therapy

Convalescent plasma (CP) therapy is another potential treatment for COVID-19. With rates of infection rising and no particular therapy available, CP therapy has been suggested as a primary treatment (188). In this treatment, to develop instant humoral immunity against SARS-CoV-2, patients are administered with plasma collected from a recovered donor. The plasma from the donor patient serves as an anti-infection reservoir of human antibodies. CP has a wide immunomodulatory effect on many molecules, including anti-inflammatory cytokines and coagulation factors, contributing to an increase in the extreme inflammatory response caused by viral infection. Such passive immunotherapy is capable of exerting both active inflammatory cytokines neutralization and passive viral neutralization, thereby inducing immunomodulation, reduced inflammation, inflammation-associated injury and viral load. Large-scale clinical trials, however, need to be performed to better understand and evaluate CP as a COVID-19 treatment tool (189).

Intravenous immunoglobulin (IVIG) is a form of immunotherapy that is made from the plasma of thousands of healthy donors and is used to treat a variety of autoimmune and inflammatory diseases. A recent open-label study in three patients identified benefits of IVIG therapy (0.4 g/kg for 5 days) in serious SARS-CoV-2-induced pneumonia, thereby offering a further alternative for the treatment of COVID-19 patients. A systematic analysis of 58 serious or seriously ill cases of COVID-19 has showed that adjunct IVIG therapy decreased hospital stay and ventilation and increased 28-day survival within 48 h of hospitalization (149). Although these studies provide a guideline for initiating a randomized clinical trial with a large number of patients some key aspects need to be considered. First, it could be prohibitively expensive, and second, the need to save the lives of primary immunodeficient patients should be evaluated. There is currently an IVIG scarcity worldwide, and the COVID-19 pandemic would disrupt the collection of plasma from donors for IVIG processing. As a result, delivering IVIG to those patients who depend on it should take precedence over starting an IVIG trial in COVID-19 patients during this emergency phase (190). Numerous case-reports explaining the efficacy of IVIG against SARS-CoV-2 are available in the literature; nevertheless, it is very difficult to gather solid data from them on the basis of various IVIG formulations and doses, patient comorbidities and quality of treatment.

SARS-CoV-2 glycoproteins can also be neutralized by the use of monoclonal antibodies (mAbs) to target the trimeric S protein on the surface that mediate entry in host cells. Identifying a human mAb that neutralizes SARS-CoV-2 is critical (163). These cross-neutralizing antibodies may attack these viruses as a common epitope and provide the potential for COVID-19 prevention and care. Tai et al. established that the SARS-CoV-2 S RBD bound strongly to human and bat ACE2 receptors (191, 192). Wang et al. reported a 47D11 (human) mAb neutralizing SARS-CoV-2. Authors found that the 47D11 attaches a conserved epitope of the spike-RBD and cross-neutralizes SARS-CoV-2. These neutralizing antibodies may minimize virus activity or protect a virus exposed uninfected host (72). Targeting RBD amino acid can help scientists create successful therapeutic agents to prevent and mitigate SARS-CoV-2 infection. These antibodies could provide an immediate strategy for emergency prophylaxis and SARS-CoV-2 treatment, whilst alternate, time-consuming vaccination and new medications are ongoing (193). However, the intravenous administration of mAbs is costly, forcing this type of therapy to be available only in high-income countries (194). Besides standard antibodies, camelids produce heavy-chain antibodies (HCAbs) consisting of just two heavy chains with a single variable domain (VHH or nanobody) and two constant regions per chain. Nanobodies (nAbs) have some special features relative to standard antibodies owing to their limited size, including access to more epitopes, low processing costs, and the prospect of large-scale development in prokaryotic expression systems. In addition, nAbs may be directly delivered to the site of the infection by an inhaler which is especially helpful in the treatment of respiratory diseases. The drawbacks of using nAbs as therapeutics may be that they may exhibit immunogenicity due to camel derivation and lack Fc-mediated effector functions. Humanizing and producing entirely human antibodies, however, may strengthen nanobodies. Recently, a SARS-CoV RBD-driven single-domain antibody, VHH-72, demonstrated cross-reactivity with SARS-CoV-2 RBD and was able to inhibit RBD-receptor-binding interactions. Also, a bivalent VHH-72-Fc construct demonstrated neutralizing effect against SARS-CoV-2 S pseudoviruses (195). Both antibodies may be attractive options and are less immunogenic than camelid or humanized nanobody since they come entirely from human sequences. The scFv and Fabs have fast generation time, strong affinity to the antigen, and structural stability. ScFvs and Fabs are promising to target COVID-19 and have already shown advantages in the battle against SARS-CoV and MERS-CoV. *In vitro*, 3D8 scFv effectively inhibited SARS-CoV-2 and other coronaviruses (HCoV-OC43 and PEDV) (196). However, the *in vivo* inhibitory effect of 3D8 scFv against SARS-CoV-2 remains to be determined.

CoV have large mutation rates and NAbs have some drawbacks. The two virus-neutralizing antibodies that constitute REGN-COV2 bind non-competitively to the essential RBD of the virus S protein, which decreases the capacity of mutated viruses and protects against S variants that have emerged in the human population (197). However, the solution of cocktail therapy is expensive and cannot cause long-term immune responses. Polyclonal antibodies could also be a good option to avoid such mutation rates (198). An alternative source of hyperimmune serum is the immunization of animals. It has been proposed that avian IgY from egg yolk could be used for oral prophylaxis. The scale-up production of hens and automatization of egg processing could provide a good alternative for the COVID19 pandemic situation (199). Clinical studies are underway for the development of an intranasal anti-SARS-CoV-2 IgY (https://clinicaltrials.gov/ct2/show/NCT04567810) (200).

### Peptides With Antiviral Activity

**Figure 6:**
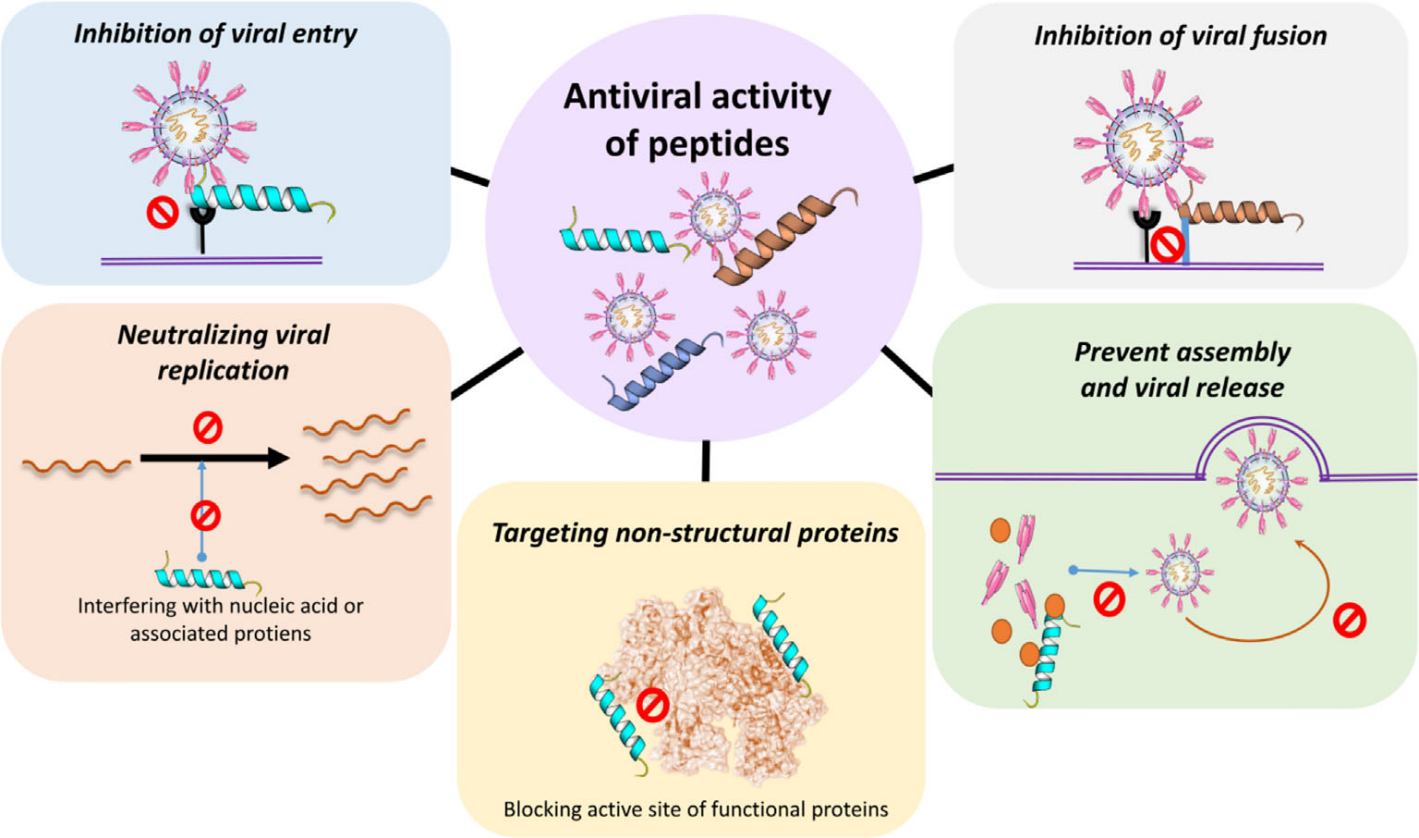
Antiviral activity of peptides. Peptides can directly prevent or inhibit the viral entry, viral fusion and or the assembly and viral release. Peptides can also neutralize viral replication by interacting with relevant proteins inside the cell. For example, blocking the active sites of functional non-structural viral proteins and/or interfering with the generation of nucleic acids directly or *via* associated proteins.

Therapeutic consistency of peptides is persuasive. Compared with chemical drugs, peptide drugs have fewer side effects and less drug resistance. The action of peptide inhibitors has been demonstrated against several viruses, including CoVs (201). During a pandemic like COVID, drug action is crucial, and peptide-based therapeutics are convincing alternatives in this regard. The advantages of using peptides are that (i) they are able to inhibit protein-protein interactions (ii) they could be used as an option for diseases that are difficult to target, (iii) it is possible to use techniques for the enhancement of peptide half-life, and (iv) they have a shorter time to market (202). Antimicrobial peptides (AMPs) are part of the first line of protection of the immune system developed by both eukaryotic and prokaryotic species. They are small gene-encoded, positively charged peptides, toxic to bacteria, protozoans, fungi and viruses (203). Their selective toxicity is due to the fact that the microbes’ bilayer membrane is rich in lipopolysaccharides (LPS) and lipoteichoic acid (LPA) and, unlike the positive charge of AMPs, is negatively charged. AMPs derived from non-virus species may have antiviral activity. However, they may have a broad spectrum of activity and exhibit other functions, such as immunomodulatory activity (204). Moreover, if the peptide produces a neutralizing epitope that can inhibit the interaction between the ACE2 receptor and the virus S protein, the epitope may be used for the generation or vaccination of an effective neutralization of antibody. For instance, specific antibodies can be produced by peptide immunization (205). Different approaches in peptide-based therapeutic development include direct interaction (“virolysis”), blockage of host cell surface receptors, inhibition of viral fusion to host cells, inhibition of viral replication and activation of adaptive immune response (202). The key mechanism of action of antiviral peptides can generally be classified into three main groups: (1) peptides that inhibit fusion (2) peptides that inhibit virus entry and (3) peptides that inhibit replication and (4) peptides that inhibit virus assembly and release (**Figure 6**).

#### Peptide Inhibiting Viral Fusion

Synthetic peptides are useful for developing effective therapeutic medicines to prevent S protein fusion and virus infection. Inhibiting peptides can establish a spatial block between S protein and ACE2 (206). A lipopeptide derivative of EK1 (EK1C4) is a highly effective inhibitor of S-protein-mediated cell-cell fusion (207). Defensins are a class of amphiphilic antimicrobial peptides. Human defensin 5 (HD5) is found in intestinal epithelial Paneth cells and granulocytes and has a strong affinity for ACE2 inhibiting viral adhesion. HD5 has the potential to also interact with SARS-CoV-2 S1, decreasing the activity of the SARS-CoV-S2 fusion protein (208). Human beta defensin 2 (hBD-2) and cathelicidin LL-37 peptoids have the distinct advantage of being insensitive to proteases, and also exhibit increased bioavailability and stability ( (209).

#### Peptide Inhibiting Viral Entry and Replication

The process of entrance and replication of CoVs in the infected host starts when the S protein on the surface of the CoV fuses with the ACE2 or DPP4 receptor to allow viral entry into the host. Mutant mucroporin-M1, a peptide derived from the parent peptide mucroporin (LFGLIPSLIGGLVSAFK) isolated from the venom of the scorpion *Lychas mucronatus*, was suggested to function as a molecular blocker that must find its target before viral attachment to host cells and demonstrated activity against SARS-CoV by inhibiting viral replication (210). Recent investigations showed that there are several potential heparin-binding sites located within the S1 domain of SARS-CoV-2. Heparin-binding peptides (HBPs) are capable of stopping SARS-CoV-2 infection (211).

EK1 (SLDQINVTFLDLEYEMKKLEEAIKKLEESYIDLKEL) is a pan-CoV fusion inhibitor that showed high cross-reactivity against all SARS-CoV, MERS-CoV, and three more SARS-related CoVs (212, 213). EK1 acts by blocking the HR1 domain to disrupt the formation of the 6HB core, which causes inhibition of viral fusion entry into the host cell (212). HCoV-OC43 alphaCoV and MERS-CoV challenges also protected mice from pre-and post-challenges. These simultaneous medicinal and prophylactic effects of one compound against multiple strongly associated CoVs are particularly effective (213). HD5 (ATCYCRTGRCATRESLSGVCEISGRLYRLCCR), a normal lectin-like human defensin-5 (HD5) peptide secreted by Paneth cells in Lieberkuhn crypts, was found to interact with glycosylated proteins and lipid components. HD5 blocked ACE2 receptors on host cells (214). The cross-linking peptide 8P9R showed potent antiviral activity against SARS-CoV-2 by cross-linking viruses to reduce viral entry on cell surface, and by interfering endosomal acidification to block viral entry through endocytic pathway (215).

#### Peptides That Prevent Assembly and Virus Release

The benefit of peptide repurposing is that for the treatment of MERS, it can contribute to the discovery of peptide-based therapeutics with possibly broader efficacy of therapeutics for the treatment of infections induced by multiple human CoVs. In addition, this will help maintain the consistency and decrease the expense of the production of new drugs. The mouse β-defensins-4 related P9 (NGAICWGPCPTAFRQIGNCGHFKVRCCKIR) was shown to bind to the MERS-CoV S2 subunit and stayed co-located with the viruses. Inside endosomes, P9’s polycationic property induced a simple microenvironment to avoid late endosomal acidification (212).

The stress-inducible molecular chaperone GRP78 can form a complex with the SARS-CoV-2 Spike protein and ACE2 intracellularly and on the cell surface and may also act as another receptor that assists SARS-CoV-2 to penetrate the host cells (216). In silico approaches have led to the identification of 5 peptides (satpdb18674, satpdb18446, satpdb12488, satpdb14438, and satpdb28899) that can block the interaction of the SARS-CoV-2 Spike protein and its binding region in GRP78 (217). However, further bioassays are needed to confirm the inhibitory activity of these compounds against SARS-CoV-2 infection.

#### Non-Structural Protein Related Peptides

Two peptides (K12 and K29) were extracted from SARS-CoV’s nsp10. These two peptides block SARS-CoV replication at an inhibitory concentration of 160 μM (214). T-cell immunity is essential to viral infection prevention. To define T-cell immunity, but also to create vaccines, it is important to recognize exact T-cell epitopes. Based on the SYFPEITHI and NetMHCpan algorithms, 1,739 and 1,591 SARS-CoV-2-derived HLA class I- and HLA-DR-binding peptides were detected across all ten viral ORFs. The proposed epitopes recognize heterologous and post-infectious T cell immunity and promote the production of COVID-19 diagnostic, preventive and therapeutic interventions (218).

## Vaccines and Vaccination

Vaccines is one of the most significant advances in human medicine for reducing the spread and effects of infectious disease. Vaccines have been used to contain epidemics and are a useful strategy in reducing pandemic mortality (219). The development of a COVID-19 vaccine has been even more rapid than that of any other vaccine. Within less than 12 months after the beginning of the COVID-19 pandemic, teams had developed vaccines that could be deployed for protection of the population from SARS-CoV-2 (220). Each vaccine that has been granted or is being considered for temporary licensing has been validated in trials involving over 20,000 patients, with months of safety evidence collected. Approximately 270 COVID-19 vaccines are presently in varying phases of production, with others using similar technology to already used vaccines and some using novel approaches (221).

An “ideal” COVID-19 vaccine should:

- elicit a strong immune response that results in long-lasting neutralizing antibodies to SARS-CoV-2 antigens.
- stimulate potent T-lymphocyte immunity to inhibit viral replication, as well as the development of memory T-cells to prevent reinfection.
- protect against both clinical illness and viral transmission, thus disrupting the process of pandemic dissemination from person to person.
- minimize any significant adverse events (SAEs) at the injection site or systemically, it is important to block adverse host responses.

Developing a safe and reliable vaccine usually takes several years since it must undergo clinical trials. However, the unexpected spread of the SARS-CoV-2/COVID-19 pandemic prompted an extraordinary attempt to develop a vaccine against this virus in a relatively limited period of time. While clinical trials have been conducted more rapidly since the pandemic, this has been accomplished by overlapping the various phases of clinical testing (phase 1, 2, and 3) rather than sequentially completing them. The safety assessment process was not affected, and the experiments adhered to the same stringent legal standards as other vaccine tests (222).

**Figure 7:**
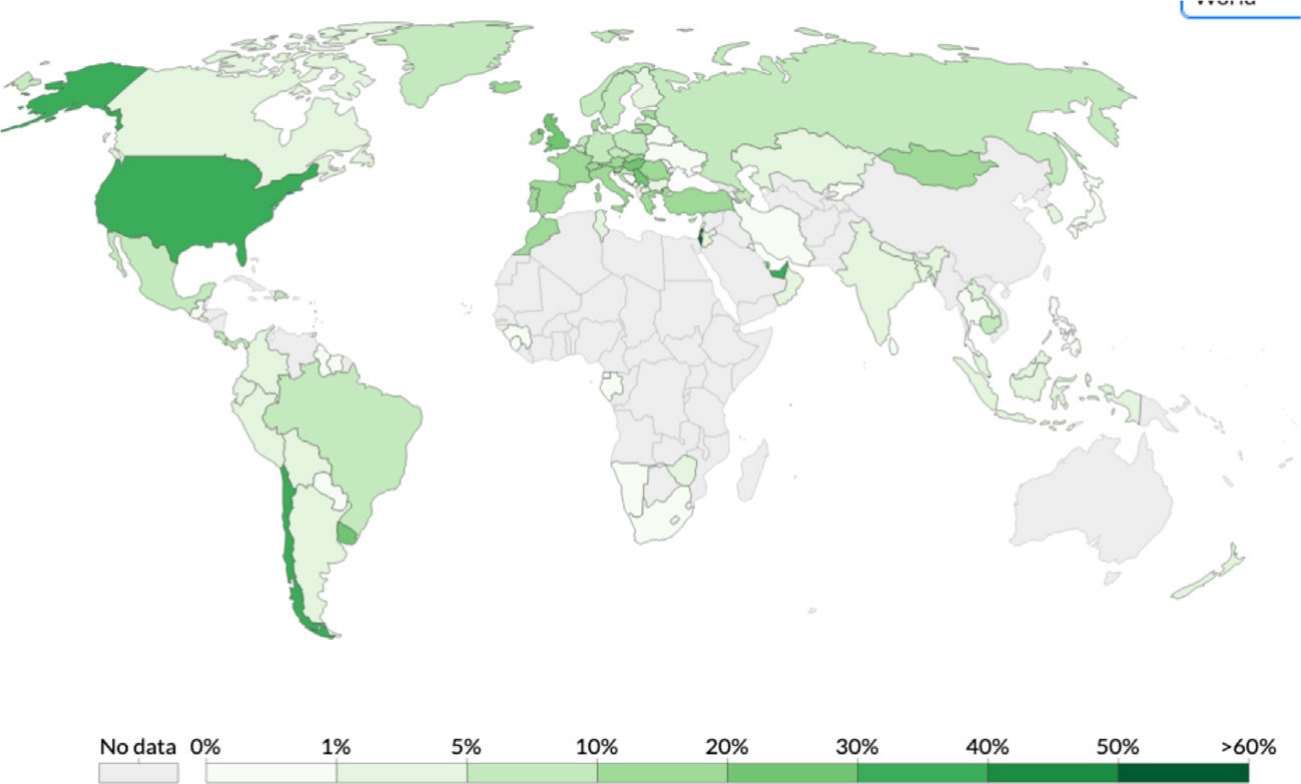
Share of the population fully vaccinated (have received all doses prescribed by the vaccination protocol) against COVID-19, May 10, 2021. Retrieved from: ‘https://covid-nma.com/dataviz/’ [Online Resource].

Before being formulated and marketed, vaccines must obtain stringent regulatory approval in the country of manufacture by a national regulatory authority (NRA). As of May 2021, about 1.3 billion vaccine doses have been distributed globally, an average of 17 doses per 100 individuals (https://www.nytimes.com/interactive/2021/world/covid-vaccinations-tracker.html). There is also a significant disparity between vaccine systems in various nations, with several countries unable to record even a single dose (**Figure 7**).

Israel was the first country to demonstrate that the COVID-19 vaccines had an effect on viral transmission of SARS-CoV-2 in society. By February 2021, more than 84 percent of people aged 70 and over had received two doses, making the country the global leader in vaccines. The number of severe COVID cases and deaths dropped dramatically (223). Related findings were seen in a separate study conducted in the United Kingdom (224).

Although vaccines still remain scarce worldwide, the majority of countries have concentrated their early vaccination programs on high-risk populations such as the clinically vulnerable; people in their 60s, 70s, and older; and front-line staff such as doctors and nurses (222).

At this stage, few adverse effects have been recorded from SARS-CoV-2 vaccine clinical trials, and the efficacy of vaccines that have been extensively tested seems to be encouraging. The duration of vaccine immunity is vital to maintaining herd immunity, estimated at 50% to 67%, without population immunity, in the absence of intervention and considering all individuals similarly vulnerable and contagious (225). In the event that COVID-19 vaccinations become more widely available, vaccination programs could expand to include younger people in order to address asymptomatic transmission, which contributes significantly to the dissemination of SARS-CoV-2 and is more prevalent in younger age groups, thus restricting the virus’s dissemination and potentially protecting the most vulnerable people (222, 225).

Another significant issue with vaccines is manufacturing capability. Given that billions of people will be vaccinated, it appears that only 2-3 companies will be able to satisfy this demand. In addition to the vaccine supply problem, logistical issues have also evolved significantly. In terms of logistics, the optimal vaccine for COVID-19 should:

- be simple to deliver, ideally in a single dose of the smallest volume available.
- be simple to manufacture and scale-up.
- be simple to transport and store.

### Vaccine Platforms

**Figure 8:**
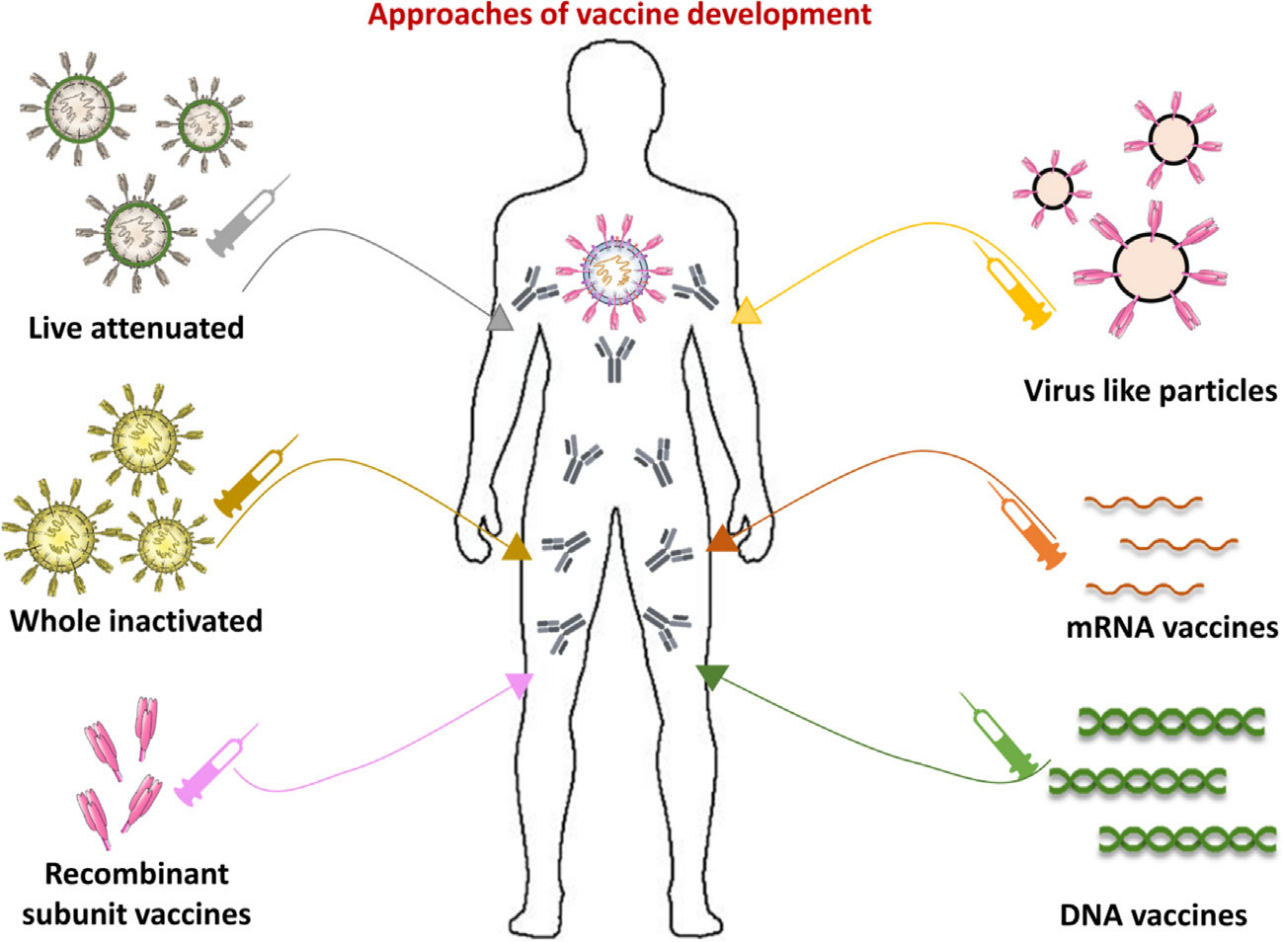
Approaches of vaccine development. Current anti-viral vaccine strategies can be divided into those that resemble the virus and molecular-based or targeted vaccines. The vaccines based on attenuated or inactivated virus, and virus like particles (VLPs) keep viral structures, and or main components, whereas molecular targeted vaccines are composed on recombinant subunit proteins, and/or nucleic acids, such as DNA or RNA. A complete list of vaccines in development for each platform can be found at https://www.covid-19vaccinetracker.org.

Aside from infrastructure, there are a range of novel vaccine innovations that are shortening the period between formulation, preclinical testing, and structured clinical trials (226). A striking aspect of current COVID-19 vaccine production system is the heterogeneity of technologies being tested, including protein, nucleic acids and live attenuated vaccines (**Figure 8**). According to the COVID19 vaccine tracker (https://www.covid-19vaccinetracker.org) a total of 257 vaccines are currently in development, and 78 are in clinical testing. Different models of vaccine platforms ensure that a wide range of alternatives to treatment are possible (226, 227). Most COVID-19 vaccine candidates are designed to trigger anti-viral S protein neutralizing antibodies, preventing uptake through the human ACE2 receptor and thereby blocking infection (228).

#### Nucleic Acid-Based Vaccines

Nucleic acid vaccines (DNA or RNA-based) transmit genetic instructions, primarily for the expression of virus spike protein, into the human cell. Nucleic acid messenger RNA is an intermediary between coding DNA and protein synthesis in the cytoplasm. Interest in these vaccines has evolved as a result of their ability to be manufactured in the laboratory using readily available materials, without the need of any culture or fermentation. This enables the procedure to be streamlined and scaled up, resulting in a faster development of vaccines than might be conceivable by traditional approaches. In terms of its high promise, capacity to evolve efficiently and cost-effectively mRNA vaccines offer a viable option to traditional vaccines (229).

#### Inactivated Vaccines

Inactivated vaccines are created by inactivating viruses with chemicals, UV light, or heat. Inactivated vaccines are desirable because they present several immune-recognition viral proteins, have stable expression of conformation-dependent antigenic epitopes and can be efficiently generated in large quantities, compared to other vaccine forms. However, since the virus must be grown in the lab before being inactivated, inactivated vaccines require longer time to produce. Traditionally used in vaccine production, purified inactivated viruses have been shown to be successful at combating infectious diseases such as influenza. Unlike their live attenuated counterparts, these inactivated vaccine viruses are not transmissible. A benefit is that they can be safely prescribed to those individuals with compromised immune systems. These vaccines, on the other hand, do not elicit a cell-mediated immune response and need several booster doses (230).

#### Vector Based Vaccines

Viral-vector based vaccines uses viral backbones, such as adeno or pox virus, to insert a SARS-CoV-2 gene into the host organism. The vaccine against viral vectors is a promising prophylactic approach. These vaccines transmit genes to target cells in a particular manner, are highly efficient in gene transduction, and elicit an immune response. The main benefit of vector vaccines is that the immunogen is expressed in a heterologous viral infection that induces the innate immune responses required for adaptive immune responses. Viral vector vaccines have a longer half-life and higher antigenic protein expression, and therefore have a greater prophylactic application because they enable cytotoxic T cells, which contribute to the elimination of virus-infected cells. Replicating vectors (measles virus and vesicular stomatitis virus) and non-replicating vectors (Adenoviruses (Ad) and poxviruses) are the two types of viral vectors. A non-replicating Ads vector is used in the manufacture of many vaccines. These vectors are physically and genetically intact, and they do not merge into the host genome. Ad vectors, on the other hand, require large doses to elicit an immune response from the host (231)

#### Subunit and Virus-Like Particles Vaccines

Subunit vaccines may contain immunogenic antigens that can stimulate the host immune system. Several antigens have been considered as promising for the development of subunit vaccines: vaccines based on full-length S protein, (RBD-Based) subunit vaccines and vaccines based on S2 subunit. The RBD-based subunit vaccine could be the best and safest option for developing a CoVs vaccine since the RBD of SARS-CoV-S comprises major antigenic epitopes that elicit both neutralizing antibodies and T cell responses (83).

VLPs are another form of protein-based vaccines consisting of viral capsid proteins. VLPs have a conformation that is similar to that of native viruses. VLPs include nearly all viral proteins but lack the viral genome and non-structural proteins that cause disease transmission. VLPs cannot reproduce in the host due to a lack of genetic content, however they may express both cellular and humoral immune responses (232).

### Leading Vaccines

As of May 2021, Fourteen COVID-19 vaccines have been given use authorizations by national regulatory authorities (https://www.covid-19vaccinetracker.org), 8 vaccines are approved for full use and six vaccines in early or limited use (**Table 1**).

**Table 1:** Authorized or approved vaccines as of April 2021.

Vaccine name	Type	Countries approved	Efficacy
Cominarty	mRNA	Bahrain, Brazil, New Zealand, Saudi Arabia, Switzerland, WHO Countries	91%
Moderna	mRNA	Switzerland, WHO Countries	>90%
Vaxzevria	Vector	Brazil	76%
Johnson & Johnson	Vector	Bahrain, Brazil, Canada, Colombia, European Union, Denmark	64-72%
Sputnik V	Vector	Russia	92%
Sinovac	Inactivated	China	50-91%
Convidecia	Protein	China, Chile, Hungary, Mexico, Pakistan	65%
Covaxin	Inactivated	Botswana, Guatemala, Guyana, India, Iran, Mauritius, Mexico, Nepal, Nicaragua, Paraguay, Philippines, Zimbabwe	78%
EpiVacCorona	Peptide	Turkmenistan Russia	Unknown
ZF2001	Protein	China, Uzbekistan	Unknown
BBIBP-CorV	Inactivated	Bahrain, China, United Arab Emirates	78%
Sinopharm	Inactivated	China	Unknown
QazVac	Inactivated	Kazakhstan	Unknown
CoviVac	Inactivated	Russia	Unknown

Source: https://www.nytimes.com/interactive/2020/science/coronavirus-vaccine-tracker.html.

https://www.raps.org/news-and-articles/news-articles/2020/3/covid-19-vaccine-tracker.

Emergency use. Early use. Stopped use.

#### Comirnaty

**Figure 9:**
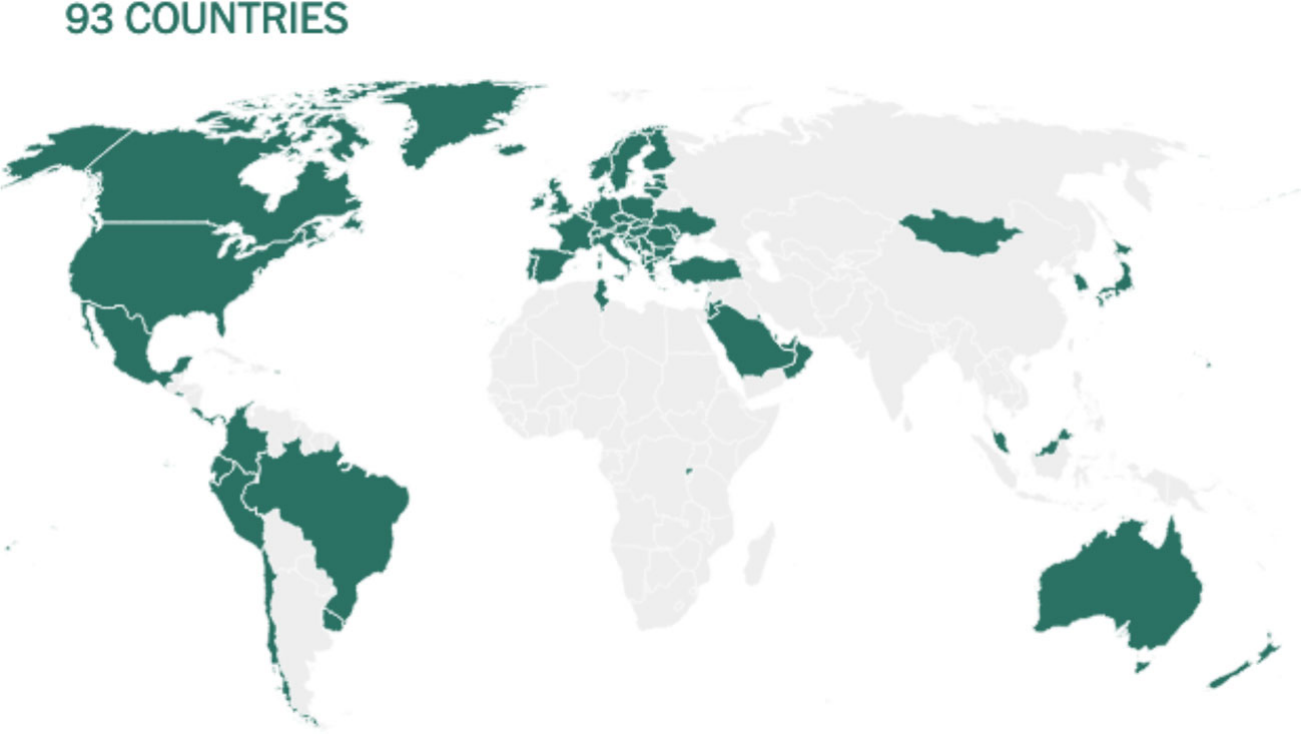
Countries where the Cominarty (Pfizer-BioNTech) vaccine is being used as of May 10, 2021. Source: Our World in Data (https://ourworldindata.org/).

Comirnaty (also known as tozinameran or BNT162b2) uses a relatively new technology: it comprises an mRNA that codes for the virus’s spike protein encapsulated in a lipid nanoparticle. Once delivered into the body by muscle injection, the host cells produce the spike antigen, alerting the body’s immune system to trigger an immune response. The body starts the production of antibodies and the formation of memory cells. Comirnaty is administered in 2 doses, 3 weeks apart. In Phase III trials, the vaccine showed 95% efficacy and a similar effect across a wide range of individuals and factors, such as age, height, race, ethnicity, and body mass index or prevalence of other diseases (233). On rare occasions (11 cases in 18 million vaccinations) mRNA vaccines caused anaphylaxis, a serious reaction that can be treated with epinephrine. As a result, the Centers for Disease Control and Prevention (CDC) requests that everyone be monitored for 15 minutes after receiving the COVID-19 injection, and for 30 minutes if they have a history of serious allergies or are taking a blood thinner (234). On 21 December 2020 Comirnaty received a conditional marketing authorization valid throughout the EU. The company is now testing in children as young as 6 months. On May 2021, the European Medicines Agency (EMA) has recommended a change to the approved storage conditions of Cominarty that will facilitate the handling of the vaccine in vaccination centers across the European Union (EU). This change extends the approved storage period of the unopened thawed vial at 2-8°C from five days to one month. As of May 2021, Cominarty is being used in 93 countries (**Figure 9**).

#### Moderna

**Figure 10:**
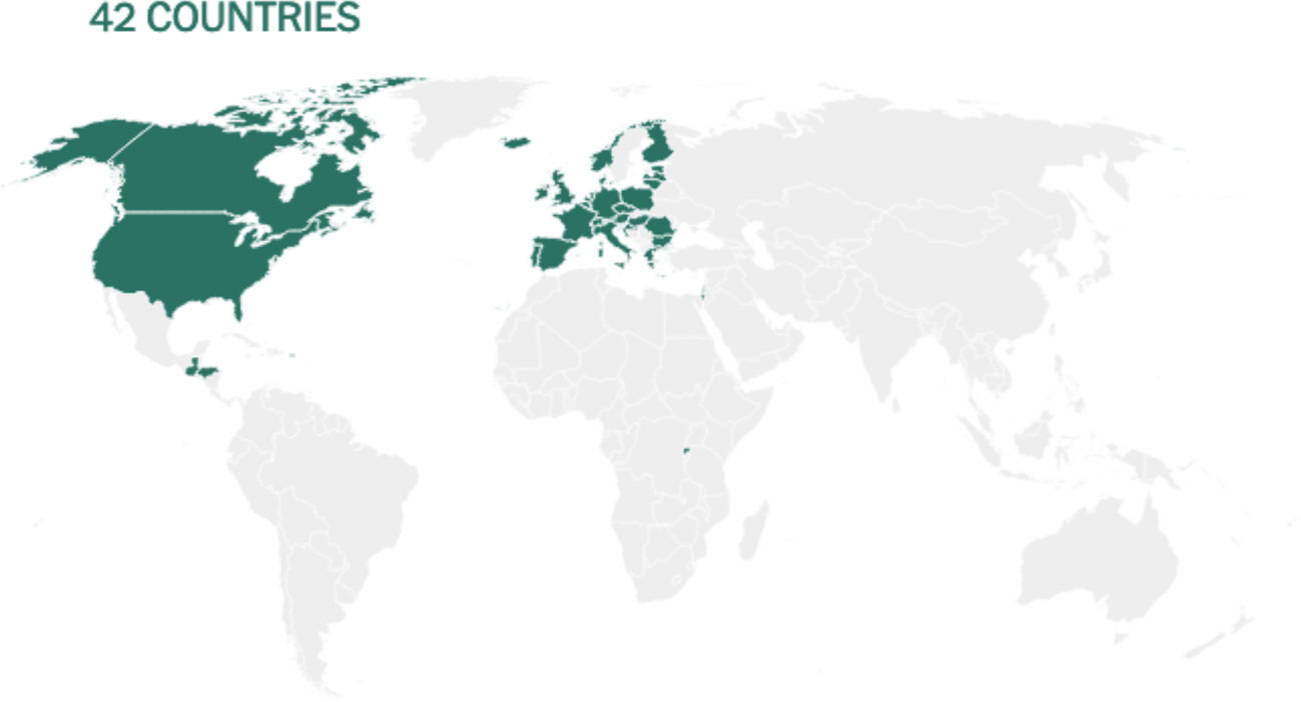
Countries where the Moderna vaccine is being used as of May 10, 2021. Source: Our World in Data (https://ourworldindata.org/).

Also known as mRNA-1273, it is an mRNA vaccine, similar to the Cominarty vaccine and has a high effectiveness in preventing symptoms. There are two significant differences: the Moderna vaccine can be delivered and can be frozen at regular freezer temperatures for up to 30 days, making it easier to deploy and stock. In addition, in clinical trials, the Moderna vaccine was marginally less effective (around 86 percent) in people aged 65 and over. Moderna is given as two injections, usually into the muscle of the upper arm, 28 days apart. On November 16, Moderna released a preliminary data readout for its COVID-19 vaccine, indicating a 94.5 percent efficacy (235). The FDA approved it on December 19, 2020. On 6 January 2021, Moderna received a conditional marketing authorization valid throughout the EU. Similar to the Cominarty vaccine, side effects can include chills, headache, pain, tiredness, and/or redness and swelling at the injection site, all of which generally resolve within a day or two (236). COVID vaccines and safety: what the research says. Nature, 590(7847), 538-540). Approved for use in Switzerland, Moderna is still testing the vaccine in children ages 12-17, and children aged 6 months to 12 years and it is currently being used in 42 countries (**Figure 10**).

#### Vaxzevria

Vaxzevria was created in early 2020 by Oxford researchers. It is made up of a virus of the adenovirus family that has been modified to contain the spike protein’s genetic material. The vaccine developed by AstraZeneca and the University of Oxford is based on technologies developed by Vaccitech, an Oxford spinout corporation. It uses a replication-deficient viral vector derived from a weakened version of a common cold virus (adenovirus) that infects chimpanzees. After being vaccinated, the cells produce the spike antigen, increasing the immune system’s anti-defense capabilities against SARS-CoV2.

On 29 January 2021, AstraZeneca received a conditional marketing authorization valid throughout the EU. Vaxzevria updated its data analysis of its phase 3 trials in March 2021, showing its vaccine to be 76% effective at reducing the risk of symptomatic disease 15 days or more after receiving the two doses, and 100% against severe disease. The company also said the vaccine was 85% effective in preventing COVID-19 in people over 65 (237).

AstraZeneca vaccine can be stored, transported and processed for at least six months under standard cooling conditions and is administered in current healthcare environments, at 2°-4°C. It can be used much more extensively than mRNA vaccinations because it only has to be refrigerated rather than frozen. However, Vaxzevria trajectory has been tumultuous, jolted by conflicting messages from AstraZeneca, high-profile safety concerns, and manufacturing difficulties (237). In April 2021, the European Medicines Agency’s (EMA) protection committee concluded that “unusual blood clots and reduced blood platelets should be identified as extremely unlikely side effects” that could occur within two weeks of vaccination. Although the UK has requested further investigation, EMA regulators have stated that the vaccine’s benefits continue to outweigh the risks. More and more countries are stopping use, or restricting the use to elderly. Typically, it is regarded that younger has a higher probability of contracting VITT than suffering from severe COVID-19. As such, use is more and more restricted to the age groups more at risk for Covid-19

**Figure 11:**
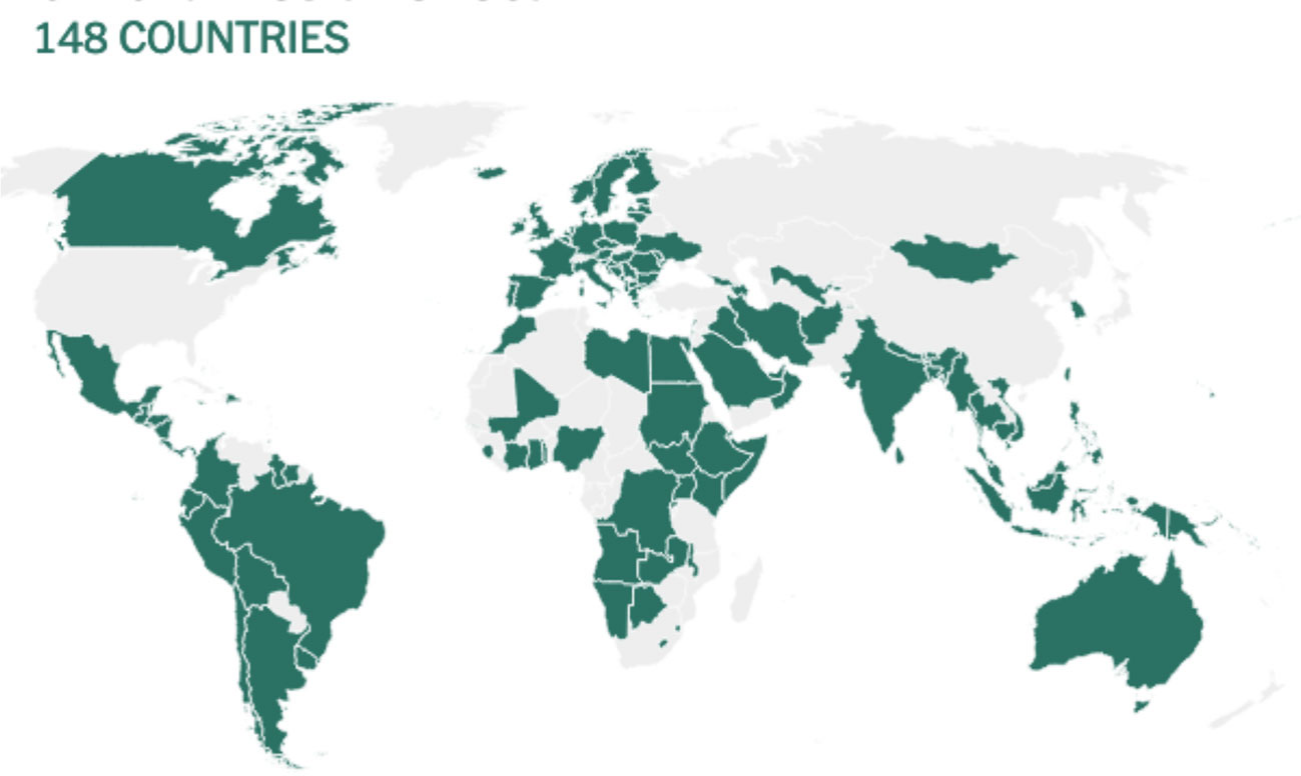
Countries where the Vaxzevria (Oxford-AstraZeneca) vaccine is being used as of May 10, 2021. Source: Our World in Data (https://ourworldindata.org/).

Vaxzevria vaccine is currently approved for use in Brazil, and it is being administered in 148 countries (**Figure 11**). It was stopped to use in Denmark.

#### Ad26.COV2. S

**Figure 12:**
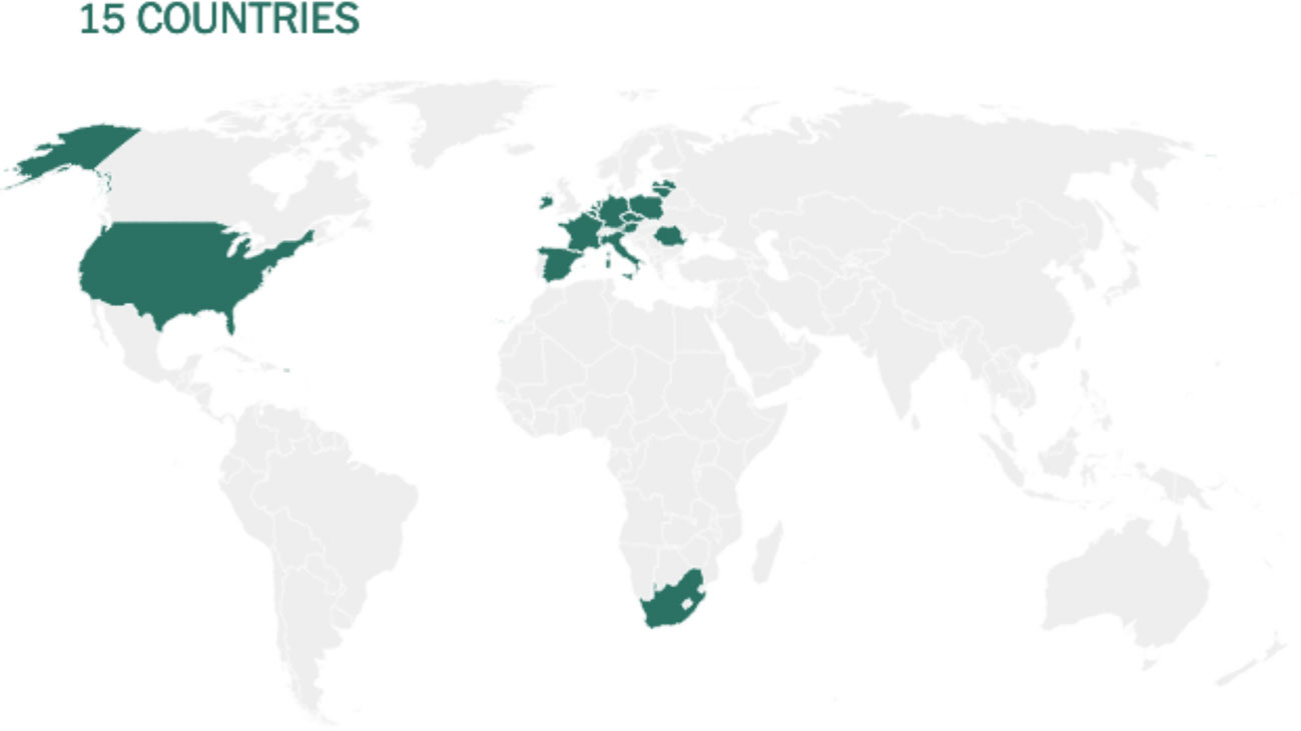
Countries where the Ad26.COV2. S-Jansen (Johnson&Johnson) vaccine is being used as of May 10, 2021. Source: Our World in Data (https://ourworldindata.org/).

Also known as Janssen, the Ad26.COV2. S vaccine is composed of another virus, of the adenovirus family, that has been modified to provide the gene responsible for producing the SARS-CoV-2 spike protein. The vaccine is based on the company’s AdVac technology platform, which was used to produce the company’s licensed Ebola vaccine as well as its investigational Zika, RSV, and HIV vaccine candidates. It is based on the use of an inactivated common cold virus, similar to the one used by AstraZeneca and the University of Oxford. The adenovirus inserts the SARS-CoV-2 gene into the cells of the vaccinated individual. The host cells would then be able to use the gene to generate the spike protein. The immune system will recognize the spike protein as foreign and will develop antibodies as well as T cells. The vaccine showed 72% overall efficacy and 86% efficacy against severe disease in the U.S. The protection starts around 14 days after vaccination but it is not known how long protection continues (237, 238). In contrast to the Pfizer and Moderna vaccines, this vaccine is easier to store and only includes a single dose, which simplifies distribution and administration. On 11 March 2021, Janssen Ad26.COV2. S vaccine received a conditional marketing authorization valid throughout the EU. In April 2021, the CDC and FDA recommended that Johnson & Johnson suspend delivery of the COVID-19 vaccine. Six cases had been reported with a “rare and severe” form of blood clot. Clots associated with the Ad26.COV2. S vaccine were cerebral venous sinus thrombosis (CVST) in association with reduced platelet counts, a condition known as thrombocytopenia. All six cases were among women aged between 18 and 48, and the single-dose vaccine occurred six to 13 days after receiving it. These six instances were exceptionally uncommon, occurring in the course of over seven million doses given (237, 238). The number of cases has since increased, and it is too soon to tell what the prevalence of VITT will be ultimately. On Friday April 23, the Food and Drug Administration (FDA) ended its recommended pause on the vaccine. Although stopped use in Denmark, Jansen vaccine is approved to use in 15 countries (**Figure 12**).

#### Sputnik V

Gamaleya produced the Sputnik V vaccine, initially called Gam-COVID-Vac. This vaccine comprises a combination of two adenoviruses called Ad26 and Ad5, rather than a standard serotype. Ad26 is offered in the first dose, followed by Ad5 in the second dose after 21 days. This technique has the benefit of producing antibodies against the Ad26 serotype after the first dose. The second dose is of the Ad5 serotype, which stimulates the body to develop a stronger immune response. Heterological vaccination can be a reasonable choice for antagonizing the harmful consequences of the immune reaction to vaccine vectors (237, 238). The Lancet published the Phase III results showing a 91.6 percent efficacy against the virus’s initial strain on February 2, 2021.

**Figure 13:**
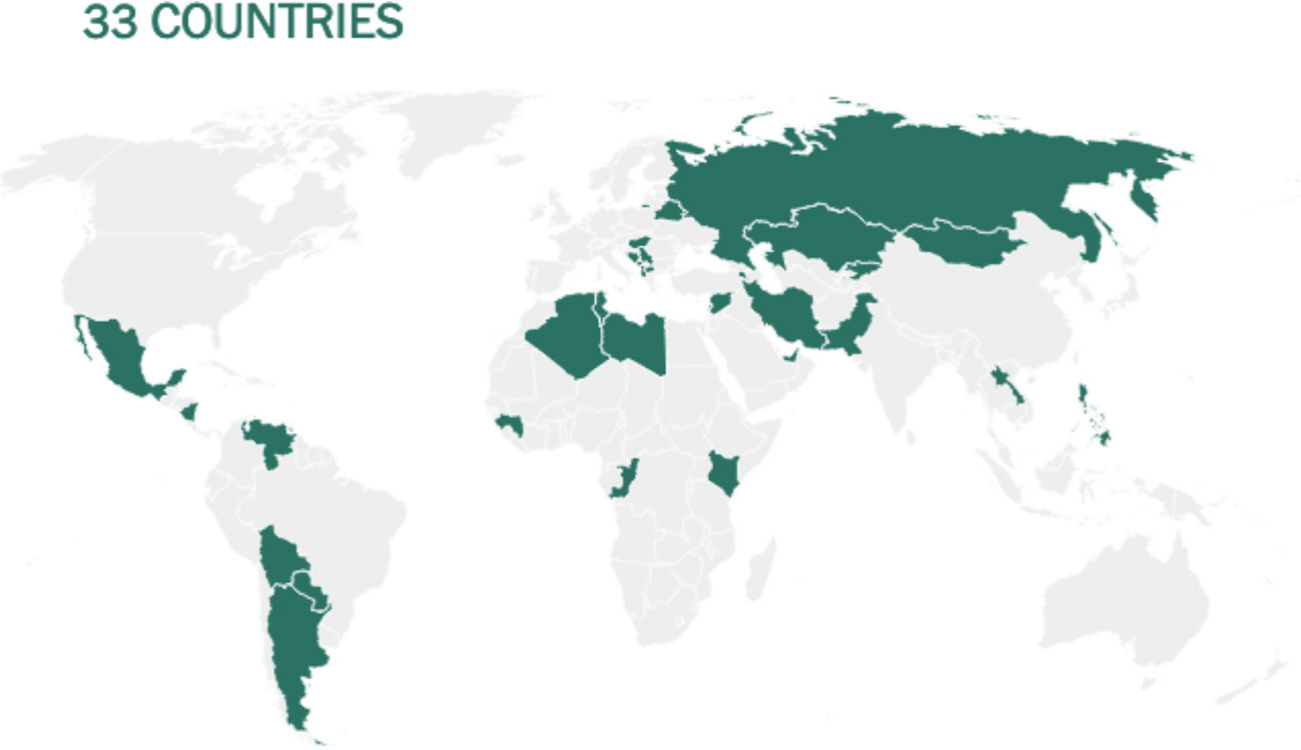
Countries where the Sputnik V (Gamaleya) vaccine is being used as of May 10, 2021. Source: Our World in Data (https://ourworldindata.org/).

Following the launch of Sputnik-V, the Russian government issued provisional requests from 20 countries for more than 1 billion doses of Sputnik V vaccine (**Figure 13**). Russia is now producing over 500 million doses of the Sputnik V vaccine (237, 238).

#### CoronaVac

In early 2020 Sinovac Biotech, a private Chinese company, developed an inactivated vaccine called CoronaVac, formerly PiCoVacc. It is administered by muscle injection in two doses, 2 weeks apart. In early 2021, trials in Brazil and Turkey showed that it could protect against COVID-19, but they delivered strikingly different results — in part because they designed the trials differently (237, 238). In Brazil, the efficacy against Covid-19 with or without symptoms was 50 percent. In Turkey, the efficacy against Covid-19 with at least one symptom was 91.25 percent. While the overall efficacy of the vaccine was lower in the Brazil trial, it showed stronger protection against severe forms of the disease. No one in the Brazil trial who received Sinovac had to be hospitalized (237, 238).

**Figure 14:**
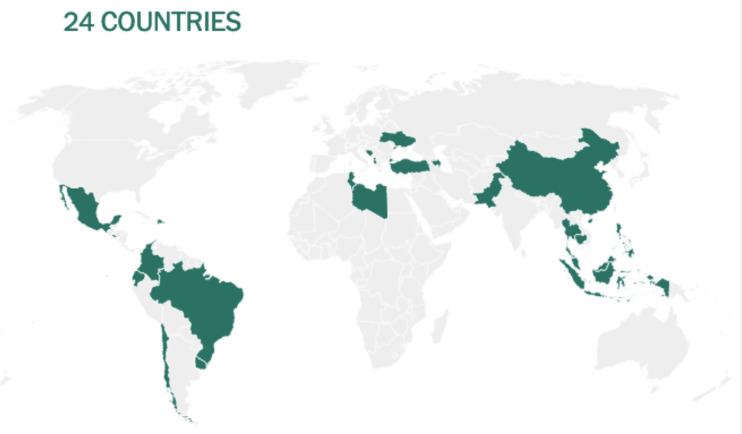
Countries where the CoronaVac (Sinovac) vaccine is being used as of May 10, 2021. Source: Our World in Data (https://ourworldindata.org/).

Sinovac released some of their data on April 3, but has yet to publish the details of the trials as a preprint or in a medical journal. Nevertheless, Sinovac announced on Feb. 6 that China had given CoronaVac conditional approval. Other countries are also beginning to use the vaccine (**Figure 14**).

Chinese government gave CoronaVac an emergency approval for limited use. In October 2020, authorities in the eastern Chinese city of Jiaxing announced they were giving CoronaVac to people in relatively high-risk jobs, including medical workers, port inspectors and public service personnel (237, 238). Sinovac has struck deals with at least 11 countries and regions to supply them with CoronaVac (**Figure 14**). On May 4, the European Medicines Agency said it was launching a rolling review of CoronaVac, which will accelerate Sinovac’s marketing authorization.

## Conclusions and Future Prospective

COVID-19 triggered by SARS-CoV-2 is a serious danger to public health. The positive cases of COVID-19 are rising at an unprecedented pace worldwide and is a matter of international concern. It is anticipated that the COVID-19 pandemic would greatly threaten to inflict immense mortality and morbidity burdens while significantly affecting populations and economies globally. In an attempt to secure billions of vulnerable individuals globally, innovative scientific research efforts are undertaken in parallel to drive forward preventive modalities. The elucidation of the SARS-CoV-2 immune defense will accelerate the development of therapeutic strategies. Particularly, the repurposing of anti-CoV and antiviral drugs with peptides already clinically approved could serve as an example for the creation of new effective and safe drugs. The urgent demand for an effective anti-SARS-CoV-2 drug could reshape the drug development landscape. As such, cocktail therapy using a selected mixture of antiviral peptides, antibodies in combination with other groups of antiviral agents could be a potential therapeutic approach that merits further clinical testing. Peptides have been formed as effective keys to viral disease. When aided with computational biology, the design of peptide therapies against SARS-CoV-2 could be accelerated more efficiently.

Following a year of global initiatives, significant progress has been made in the development of COVID-19 vaccines, and several vaccine candidates based on mRNA technology or a virus vector platform have been developed and demonstrated protective efficacy against SARS-CoV-2. To date, the manufacture of mRNA vaccines for the prevention of infection with the SARS-CoV-2 coronavirus has been a success story. However, due to manufacturing and transportation restrictions in many remote regions, vaccines produced so far are unable to fulfill global vaccination criteria. The long-term effectiveness of these vaccines, as well as associated safety issues, are being investigated. Governments, public health sector authorities and advocacy groups must be prepared to ensure access to and delivery of COVID-19 vaccines on a massive scale, in a reasonable manner. Since the leading candidates are administered intramuscularly, the emphasis is on immune responses in the blood rather than on mucosal surfaces. However, mucosal immunity plays an important role, and many intranasal vaccine formulations are being developed. An important question to be addressed is how long defensive immunity can persist after vaccination. The “long defensive immunity” post vaccination is more likely related to the emergence of new viral variants as opposed to the duration of vaccine efficacy. As is also the case for influenza.

Increased viral replication provides further chances for SARS-CoV-2 variants to evolve. As a result, vaccine production may be hampered if the virus later develops resistance to the spike glycoprotein used to create the vaccine. Vaccinations may be engineered to attack multiple viral locations, minimizing the chance of a mutated virus evading existing immunity. Despite these limitations, we will now have a completely different vaccination strategies that can be implemented rapidly the next time we are threatened with a zoonotic epidemic.

## Author Contributions

UA: Conceptualization, Literature survey, Writing major original draft, Figure designing, References collection, Review structure. SJ: Literature survey, Writing- review and editing, Figure designing. OI: Literature survey, Writing- review and editing, Figures designing. HC: Reviewing and editing, Supervision. Z-SC: Review and editing, Response. VT: Conceptualization, Critical review and editing, Review structure, Response, Supervision. JMPL: Conceptualization, Literature survey, Writing, Figures designing, Table preparation, Critical review and editing, Response. All authors contributed to the article and approved the submitted version.

## Funding

This work was funded by “Agencia Canaria de Investigación, Innovación y Sociedad de la Información (ACIISI) del Gobierno de Canarias”, project ProID2020010134 “Bioprospección y biotecnología en el descubrimiento de péptidos antimicrobianos contra patógenos resistentes” and CajaCanarias, project 2019SP43. The funders had no role in the design of the study; in the collection, analyses, or interpretation of data; in the writing of the manuscript, or in the decision to publish the results.

## Conflict of Interest

The authors declare that the research was conducted in the absence of any commercial or financial relationships that could be construed as a potential conflict of interest.
